# Advanced Neuromorphic Applications Enabled by Synaptic Ion‐Gating Vertical Transistors

**DOI:** 10.1002/advs.202305611

**Published:** 2024-05-17

**Authors:** Leandro Merces, Letícia Mariê Minatogau Ferro, Ali Nawaz, Prashant Sonar

**Affiliations:** ^1^ Research Center for Materials Architectures, and Integration of Nanomembranes (MAIN) Chemnitz University of Technology 09126 Chemnitz Germany; ^2^ Center for Sensors and Devices Bruno Kessler Foundation (FBK) Trento 38123 Italy; ^3^ School of Chemistry and Physics Queensland University of Technology (QUT) Brisbane QLD 4000 Australia; ^4^ Centre for Materials Science Queensland University of Technology 2 George Street Brisbane QLD 4000 Australia

**Keywords:** artificial synapses, brain‐inspired, electrochemical, field effects, human‐machine interfacing, multi‐modal, sensors

## Abstract

Bioinspired synaptic devices have shown great potential in artificial intelligence and neuromorphic electronics. Low energy consumption, multi‐modal sensing and recording, and multifunctional integration are critical aspects limiting their applications. Recently, a new synaptic device architecture, the ion‐gating vertical transistor (IGVT), has been successfully realized and timely applied to perform brain‐like perception, such as artificial vision, touch, taste, and hearing. In this short time, IGVTs have already achieved faster data processing speeds and more promising memory capabilities than many conventional neuromorphic devices, even while operating at lower voltages and consuming less power. This work focuses on the cutting‐edge progress of IGVT technology, from outstanding fabrication strategies to the design and realization of low‐voltage multi‐sensing IGVTs for artificial‐synapse applications. The fundamental concepts of artificial synaptic IGVTs, such as signal processing, transduction, plasticity, and multi‐stimulus perception are discussed comprehensively. The contribution draws special attention to the development and optimization of multi‐modal flexible sensor technologies and presents a roadmap for future high‐end theoretical and experimental advancements in neuromorphic research that are mostly achievable by the synaptic IGVTs.

## Graphical abstract

Replicating the human brain functions using electronics is not an easy feat. In this review article, L. Merces, P. Sonar, and coauthors outline the pioneering advancements and central concepts of synaptic ion‐gating vertical transistors toward improved signal processing, transduction, plasticity, and multi‐stimulus perception. These aspects provide a new vista for the development of low‐voltage multi‐modal synaptic networks for high‐end brain‐inspired bioelectronic applications.

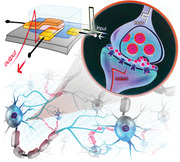

## 1 Introduction

Understanding and replicating the intricate functions of the human brain using electronics is not an easy feat. Over a century ago, Nobel Laureate neuroanatomist Santiago Ramón y Cajal discovered that the human central nervous system is composed of an intricate web of connections between countless neurons.^[^ ^1^ ^]^ The brain contains ≈100 trillion synapses, which act as bridges between one hundred billion neurons.^[^ ^2^ ^]^ These biological neural networks process information through synaptic events. Nerve impulses in the form of action potentials are generated by presynaptic neurons in response to received input or stimulus information. These impulses are then transmitted to postsynaptic neurons via the synapses. Generally, the neuron membrane devises a resting potential (from −60 to −70 mV) that is either excited or inhibited by incoming stimuli.^[^ ^3^ ^]^ Excitatory or inhibitory stimuli can create positive or negative voltage, whereas the combination of inputs beyond a certain threshold generates an action potential that controls the influx of calcium cations into the cytoplasm. This event releases neurotransmitters into the synaptic cleft. On the postsynaptic neuron, such transmitters connect to receptors, causing the ligand‐gated channel to allow sodium ions to enter the postsynaptic membrane. This transmits to the subsequent neuron the input stimuli.^[^ ^4^, ^5^ ^]^ The neurotransmitters' chemical interaction with the postsynaptic receptors changes their shape and induces succeeding reactions for ion gating. The abrupt charge disparity creates the potential that initiates another action stimulus in the postsynaptic terminal for information transmission.^[^ ^6^ ^]^ Enzymatic processes deactivate the neurotransmitters linked to the receptors, causing their departure toward the presynaptic region for the subsequent transmission.^[^ ^7^ ^]^ The synaptic weight between two neighboring neurons determines the information transmission efficiency, i.e., the memory effect. The stimuli‐responsive change in synaptic weight is known as plasticity,^[^ ^8^ ^]^ which is dependent on the amplitude and repetition of action potential spikes.

**Figure 1:**
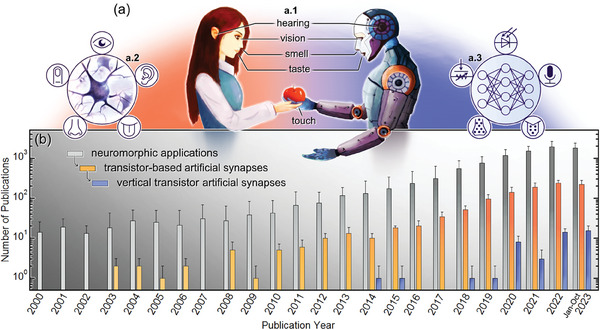
Human‐brain‐inspired multisensory functions and neuromorphic research. a) Schematic illustrations of a.1) the five primary sensory systems in the human body, a.2) multisensory functions processed by the human brain, and a.3) their emulation by artificial neural networks. Adapted with permission.^[^ ^12^ ^]^ Copyright 2021, Tan et al. Published by Springer Nature. b) The increasing number of publications regarding neuromorphic applications (viz. logarithmic scale) over the past years to October 2023. Data collected from Web of Science on November 2023. Examination fluctuations (upper error bars) arise from possible keyword combinations, article categorization, and search refinement.

In recent years, scientists have made significant progress in developing bio‐inspired synaptic systems and artificial multisensory neurons for memory and perception (**Figure** **1**a.1).^[^ ^9^, ^10^, ^11^, ^12^ ^]^ These neuromorphic devices are capable of imitating basic and advanced neural functions, such as pain perception, pattern recognition,^[^ ^13^, ^14^ ^]^ and light‐, sound‐ and pressure sensing,^[^ ^15^, ^16^, ^17^ ^]^ besides emulating the five primary human sensory systems (Figure 1a.2) via multisensory neural networks (Figure 1a.3). Among the various neuromorphic device technologies, which have led to the exponentially increasing number of publications since the 2000s, transistors have played an important role especially after 2008 (Figure 1b). Particularly, the ion‐gating transistor (IGT) has gained significant attention due to its unique features arising from its ionic‐ and/or electronic current modulation, such as high transconductance, large gate capacitance, low operating frequency, and ultrahigh carrier concentration.^[^ ^18^, ^19^, ^20^ ^]^ These properties and the IGT's structural characteristics make this device suitable for artificial synapse applications.^[^ ^21^, ^22^, ^23^ ^]^ Despite the challenges in device fabrication due to the transistor's 3‐terminal integration, the expressive current modulation enabled by ion‐gating is crucial for the simultaneous reading and writing operations required by memory.^[^ ^24^, ^25^ ^]^ Upon the application of voltage stimuli, the IGT's gate (*G*) can emulate the presynaptic function, whereas the postsynaptic response can be acquired at the source (*S*) and drain (*D*) electrodes. Multi‐gated stimulations on an IGT can be processed through this single device, providing ample room for information collection from various sources, resulting in spatiotemporal recognition apparently impossible in two‐terminal devices.^[^ ^26^, ^27^, ^28^ ^]^ Additionally, IGTs allow the integration of inorganic‐ and/or organic materials into multifunctional artificial synapses, providing versatility, mechanical flexibility, biocompatibility, and low‐cost manufacturing (e.g., printing and coating), with the ability to modify their properties via supramolecular engineering and cross‐linking.^[^ ^29^, ^30^, ^31^ ^]^

Recent research on IGTs has unveiled a game‐changer neuromorphic device − the ion‐gating vertical transistor (IGVT).^[^ ^32^, ^33^ ^]^ Accordingly, Figure 1b also showcases the number of publications focused on neuromorphic applications of vertical transistors, putting forward that research on this new device technology is just getting started. Additionally, Figure 1b indicates that vertical transistors have the potential to become one of the next exponentially rising technologies in the field of neuromorphic science. Among the vertical transistors in Figure 1b, the IGVTs are in pride of place given their distinguished mixed ionic‐electronic current modulation. Furthermore, compared with conventionally planar‐configuration devices, IGVTs display higher bias‐stress constancy, lower working voltage, and more efficient power management.^[^ ^34^, ^35^ ^]^ This is primarily due to the IGVT channel length (*L*) being determined by the *S*‐to‐*D* vertical separation, naturally, the thickness of a thin film − a feature that allows for downscaling the ionic‐electronic transport pathway to the tiny sub‐10 nm range.^[^ ^36^, ^37^, ^38^ ^]^ Secondly, whereas in conventional IGTs the lateral charge transport is more vulnerable to defects at the semiconductor/electrolyte interface, in IGVTs, the vertical charge transport significantly eliminates this side effect.^[^ ^34^ ^]^ IGVTs have indeed more outstanding mechanical stability compared to planar IGTs since the vertical charge transport enhances the semiconductor channel resilience to fissures and displacements triggered by deformation.^[^ ^39^, ^40^ ^]^ Such unique and promising features have been capturing the attention of artificial synapse research, which turned its focus recently to IGVTs. In just a few years, such devices have already demonstrated low voltage operation, low energy consumption, high recognition accuracy, and promising mechanical flexibility, multiplex integrability, and multisensory capability.^[^ ^40^, ^41^, ^42^, ^43^, ^44^, ^45^ ^]^

Our review explores the exciting potential of using vertical transistor technology to enhance ion‐gating properties in neuromorphic applications. Although there have been previous reviews that cover resembling topics related to active materials,^[^ ^46^, ^47^, ^48^ ^]^ device architectures and design principles,^[^ ^23^, ^34^, ^43^ ^]^ ion‐gating properties,^[^ ^22^, ^49^, ^50^ ^]^ and neuromorphic applications,^[^ ^35^, ^44^ ^]^ the inherent importance and novelty displayed by the use of IGVTs to boost the capabilities of state‐of‐the‐art neuromorphic applications make this review no sooner vital. In due course, here we offer an up‐to‐date overview of the latest advancements in IGVT technology, with a focus on cutting‐edge demonstrations that can revolutionize the next generation of brain‐inspired artificial synapses. We begin with an examination of the IGVT's architecture and ion‐gating principle (Section 2), highlighting the unique features that make this device ideal for high‐end neuromorphic applications. Subsequently, the topical achievements in IGVT fabrication reliability, monolithic integration processes, and electrical characteristics are discussed in Section 3, providing the standards for IGVT‐based artificial‐synapse development. Then, Section 4 brings up insightful demonstrations of neuromorphic IGVTs in high performance, high energy‐efficiency, flexible, multisensory, and multi‐modal applications. As a roadmap for advanced neuromorphic technologies, Section 5 points out the current open challenges in the field and offers our view on the most useful theoretical and experimental tools that are currently available for researchers and specialists to tackle each standing situation. Finally, Section 6 provides a critical outlook on the future of synaptic IGVTs.

## 2 Working Mechanism and Unique Features

**Figure 2:**
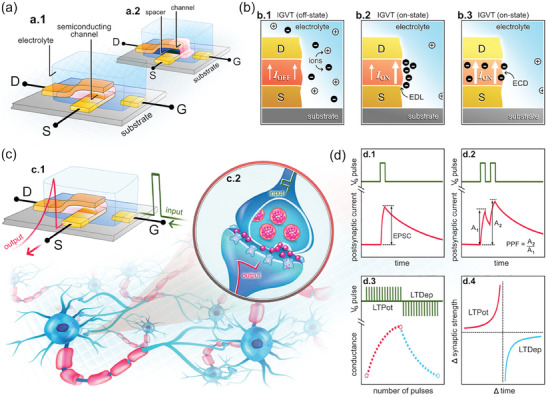
IGVT device architecture, working principle, and synaptic functions. a) Schematic illustrations of the IGVT basic structures, in which a.1) the semiconductor film is sandwiched by *S* and *D*, or a.2) a spacer layer is employed to set the *S*‐to‐*D* separation. b) Illustration of the p‐type IGVT working principle (without loss of generality). b.1) IGVT OFF‐state. b.2) IGVT ON‐state: EDL formation leading to the electric‐field effect and EG‐V(O)FET current amplification. b.3) IGVT ON‐state: ECD leading to the mixed ionic and electronic current amplification across the V(O)ECT channel. c) Artificial synapse concept. c.1) Schematic illustration of an artificial synapse based on the IGVT. c.2) Schematic illustration of a neural network and zoom in the biological synapse. d) Functions of synaptic devices. d.1) EPSC and d.2) PPF induced by single‐ and double‐pulse input stimuli. d.3) LTPot and LTDep triggered by multiple pulses. d.4) STDP in response to LTPot and LTDep.

IGVTs are vertical semiconductor channel devices based on thin‐film transistors where the semiconductor material is in contact with an electrolytic *G*, as illustrated in **Figure** **2**a.^[^ ^51^ ^]^ The IGVT electrolyte is typically based on an ionic liquid or an ionic gel.^[^ ^52^, ^53^ ^]^ The semiconductor material is normally sandwiched by *S* and *D*. This can be achieved by the materials' direct stack (Figure 2a.1)^[^ ^54^ ^]^ or assisted by the addition of a spacer layer (Figure 2a.2).^[^ ^37^ ^]^ In the former architecture, *L* is defined by the semiconductor thin‐film thickness and, in the other, it is equal to the spacer thickness. Both methods allow the production of ultra‐short *L* transistors without the need for sophisticated nanolithography techniques. However, before selecting one of the fabrication routes, it is important to consider the advantages and drawbacks of each method. The architecture displayed in Figure 2a.1 constitutes a simple solution with fewer fabrication steps. Yet, it must be considered that this method involves the deposition of *D* directly on the semiconductor film, which can influence the morphology and thus the device performance. Nevertheless, promising results have been achieved using this IGVT architecture with sub‐50 nm‐thick channels, including the demonstration of ON current (*I*_ON_) density exceeding 25 A cm^−2^ using printed single‐walled carbon nanotubes (SWNTs)^[^ ^55^ ^]^ and the efficient development of an artificial tongue.^[^ ^56^ ^]^ Comparatively, the method illustrated in Figure 2a.2 adds complexity to the fabrication process but promises further device miniaturization and performance improvement. While choosing the spacer materials, various aspects must be considered, including the cost‐effectiveness of material preparation, the material's compatibility with the preparation process, its insulation, and surface characteristics, among others.^[^ ^57^ ^]^ Keeping in view the conformity of the subsequently deposited semiconductor layer, the sidewall profile (and thus the corresponding etching process) of the spacer film must also be engineered. Most importantly, to achieve the true benefits of vertical architecture, the thickness of the spacer must be controlled with nanometer precision. This can be achieved by forming nanogaps using self‐assembled monolayers and adhesion lithography^[^ ^58^ ^]^ − which is yet to be demonstrated in IGVT applications − or by using insulating materials with atomically precise thickness and flatness. The latter has been demonstrated recently by Lenz et al. who used hexagonal boron nitride (hBN) as the spacer layer with thickness down to 2.4 nm and device output current densities as high as 2.95 MA cm^−2^ at −0.4 V.^[^ ^37^ ^]^ While the use of a spacer layer requires additional fabrication steps, it is a more reliable method that can lead to better device performance. This is because it allows for easy control of the thickness, flatness, and material properties of inorganic insulating materials. Additionally, direct deposition of *S* or *D* on the semiconductor film can be avoided (Figure 2a.2).

The current modulation in IGVTs allows the controlling of the channel transconductance so that current OFF‐ (Figure 2b.1) and ON states (Figure 2b.2,3) can be reached as a function of the applied gate voltage (*V_G_*). The IGVT working principle depends on the semiconductor's chemical structure and affinity with the electrolyte. Compacted single‐crystals of small molecule semiconductors and hydrophobic well‐organized and crystalline polymers (composed of strong π–π stacking) in aqueous electrolytes are typically ion‐impermeable.^[^ ^59^ ^]^ This situation leads to the electrolyte‐gated vertical (organic) field‐effect transistors, EG‐V(O)FETs, in which the current modulation is driven by the electric‐field effect resulting from the electrolyte ion migration to the semiconductor interface. In this scenario, a capacitive gating takes place, giving rise to the electrical double layer (EDL), as illustrated in Figure 2b.2. The EDL can be well described as a Helmholtz layer.^[^ ^51^, ^52^, ^53^, ^60^, ^61^, ^62^ ^]^ For the vertical (organic) electrochemical transistors, V(O)ECTs, the same EDL‐formation and electric‐field effect principles apply, but they are not ruling exclusively. The fundamental character of VECTs and VOECTs comes from the channel permeability to the electrolyte ions,^[^ ^63^, ^64^ ^]^ which is due to the porous structures of amorphous or semi‐crystalline semiconducting films. When such films oxidize or reduce, some anions or cations can migrate into the channel material for charge compensation.^[^ ^59^ ^]^ The ion penetration can provide the channel material with a mixed ionic and electronic charge transport, leading to the current amplification enhanced by the channel ion‐doping (Figure 2b.3).^[^ ^65^ ^]^ Thereby in VECTs and VOECTs, the current modulation is driven by the electrochemical doping (ECD) and the transport of ionic and electronic charges throughout the vertical semiconducting channel upon *V_G_* application.^[^ ^66^ ^]^ Both EDL formation and ECD provide IGVTs with higher capacitance compared to the non‐electrolytic vertical transistors. This feature added to the vertical device ultrashort *L* values, enables the IGVTs to operate at lower voltages (<1 V) and with lower energy consumption.^[^ ^67^ ^]^ Such high‐end properties make IGVTs one of the most promising candidates to drive state‐of‐the‐art neuromorphic applications toward low‐voltage, flexible, biocompatible, and fully integrated circuits within complex artificial sensor‐ and actuator systems.^[^ ^68^ ^]^

The biological synapse is composed of presynaptic and postsynaptic terminals (axon and dendrite, respectively), and the synaptic cleft. The neurotransmitter release into the synaptic cleft regulates the information transmission through the synapse. To emulate this phenomenon, the IGVT‐based artificial synapses utilize an electrolyte‐contacting *G* that imitates the presynaptic membrane of the biological axon (Figure 2c).^[^ ^69^ ^]^ The *G* terminal supplies electrical signals (input) to the artificial synapse, similar to the presynaptic spikes (Figure 2c.1). Comparable to the electrolyte role in the conventionally planar‐configuration IGTs,^[^ ^70^, ^71^ ^]^ the IGVT's electrolyte separates *G* from the semiconducting channel, emulating the synaptic cleft and providing mobile ions that migrate in response to the presynaptic stimuli (Figure 2c.2). The semiconducting channel and *S–D* electrodes mimic the postsynaptic membrane and the biological dendrite, generating response signals (output) that are analogous to the postsynaptic spikes (Figure 2c.1,2). Prior to a *V_G_* spike, the ions are randomly dispersed throughout the electrolyte. When a brief voltage pulse is applied, ions start migrating toward the semiconducting channel which further results in the accumulation of free charge carriers at the semiconducting interface or inside the channel. At a given *S–D* voltage (*V_D_*), the accumulated charge carriers promptly provide a sudden change in the channel current. This is measured at the *S–D* electrodes as the output excitatory or inhibitory postsynaptic current (EPSC or IPSC, respectively). Succeeding the presynaptic spike, ions in the semiconductor gradually return to a random distribution as there is no driving force to keep them in place. This results in a gradual decay of the postsynaptic current (PSC).^[^ ^72^ ^]^

The artificial synapse current response can be visualized in Figure 2d.1 as a function of time. Its maximum amplitude, EPSC, provides insight into the artificial synapse strength. Another fundamental synaptic function is synaptic plasticity,^[^ ^73^ ^]^ which can be categorized either as short‐ or long‐term plasticity (STP and LTP, respectively).^[^ ^74^, ^75^ ^]^ STP and LTP can be considered as the major instruments of learning and memorization processes. A typical characteristic of synaptic plasticity is the so‐called paired‐pulse response, as shown in Figure 2d.2. This phenomenon refers to the behavior where, in the course of two successive stimuli in short‐term potentiation (STPot), the EPSC caused by the last spike is superior to that caused by the earliest.^[^ ^76^, ^77^ ^]^ The ratio between the last and the earliest EPSC amplitudes (*A*_2_ and *A*_1_, respectively) is known as paired‐pulse facilitation (PPF),^[^ ^3^, ^78^ ^]^ as depicted in Figure 2d.2. When the second current peak in IPSC is decreased leading to short‐term depression (STDep), the *A*_2_/*A*_1_ ratio is equivalently referred to as paired‐pulse depression (PPD).^[^ ^78^ ^]^ The LTP behavior can be observed in artificial synapses by repetitive learning mechanism upon long‐term potentiation and depression experiments (LTPot and LTDep, respectively), as exhibited in Figure 2d.3. Additionally, IGVTs may exhibit spike‐timing‐dependent plasticity (STDP) and various types of long‐term‐memory features that depend on either spike rate or number (Figure 2d.4).^[^ ^3^, ^10^, ^69^ ^]^ As we point out in the succeeding sections, the IGVT technology can already adjust the memory plasticity strengths via either device design, heterostructure composition, or chemical, mechanical, and electronic properties of the materials.

The figure of merits for IGVTs in neuromorphic devices are not as standardized as those for traditional electronic devices and can vary based on the specific design and application. Nevertheless, some crucial considerations encompass: 1) the capability to fine‐tune the strength of connections between neurons for emulating the synaptic plasticity, 2) energy consumption per synaptic operation or per neural network inference, 3) the speed at which the transistors can adjust their weights or respond to input signals for real‐time processing and efficient operation, 4) ability to ensure the stability of synaptic weights over time, 5) a high signal‐to‐noise ratio, 6) small device footprint for constructing large‐scale neuromorphic systems, and 7) compatibility with standard fabrication processes. Among these, energy consumption (*E_c_*), which includes operating voltage, peak postsynaptic current, and spike width, can be considered one of the most important figures of merit since transistors with lower *E_c_* would contribute to aligning artificial neural networks (ANN) more closely with their biological counterparts. Lower *E_c_* enables the devices to perform complex cognitive tasks with minimal power requirements and also allows for the scalability of neuromorphic systems. As these systems grow in complexity and size, reducing the energy footprint becomes essential to ensure practical implementation and to avoid excessive power demands. Many neuromorphic applications, such as robotics and autonomous systems, require real‐time processing. Lower *E_c_* in synaptic transistors often correlates with faster operation, facilitating quick and efficient decision‐making in real‐world scenarios. Similarly, lower *E_c_* results in reduced heat generation, which allows for more compact designs and reduces the need for elaborate cooling solutions, particularly in applications where size and weight constraints are significant. More importantly, reducing the overall power consumption in neuromorphic applications aligns with sustainable practices and addresses concerns about the environmental impact of technology. In fact, it enhances the economic viability of deploying neuromorphic technology in various domains. Recently reported IGVT devices show promising performance in this regard, with both low *E_c_* as well as reduced device *L* values (**Table** **1**).

**Table 1:** List of recent works on IGVTs with short channel lengths and ultra‐low *E*_c_, showing strong compatibility with next‐generation neuromorphic applications.

Year	*L* [nm]	*V_D_* range^a)^ [*V*]	*E_c_* range [*J*]	Reference
2019	40	10^−4^	≈10^−13^	[69]
2019	55	10^−2^	≈10^−10^	[79]
2022	30	10^−4^	≈10^−17^	[56]
2022	150	10^−2^	10^−12^ – 10^−11^	[80]
2022	35	10^−3^	≈10^−13^	[81]
2023	30	10^−3^	≈10^−14^	[45]

^a)^ The *V_D_* ranges can be different from the corresponding *V_G_* ranges. Please check the references for additional details.

## 3 Architectures and High‐End Electronic Properties

Shrinking the physical size of transistors aims at better use of substrate space, also boosting device performance, and decreasing power consumption and device price.^[^ ^43^, ^82^ ^]^ Downscaling approaches for organic transistors focus on the investigation and optimization of both the morphology and charge transport properties of the organic semiconductor channel (OSC). In this scenario, the size and orientation of the semiconducting channel play an important role.^[^ ^83^, ^84^, ^85^, ^86^, ^87^, ^88^, ^89^, ^90^, ^91^ ^]^ Notably, *S–D* stacking is an alternative to obtaining transistors with short channels, higher current densities at lower working voltages, and a high integration density.^[^ ^92^, ^93^ ^]^ Section 3 summarizes the critical advances in IGVT research, from their (3.1) early‐stage investigations on architecture geometries and (3.2) electronic property characterization to their (3.3) successful achievements in IGVT logic circuits and (3.4) multi‐parametric applications. The topics discussed within these subsections (3.1–3.4) pave the way for the cutting‐edge neuromorphic applications brought to debate later in Section 4.

### 3.1 Early Realizations of IGVTs

The need for new material architectures to shorten *L* without the use of high‐end techniques is the focus of recent breakthrough research.^[^ ^37^, ^40^, ^94^ ^]^ Remarkable advancements have shown that incorporating electrolytes into vertical‐transistor architectures has numerous benefits. Among them, there is a decrease in power consumption and an increase in capacitance, both due to the dielectric based on the ultrathin EDL. The demonstration of a prototypical EG‐VOFET was provided by Liu et al. in 2010.^[^ ^32^ ^]^ Owing to the use of an electrolyte, the EG‐VOFETs with *L* in the 0.7–2.2 µm range operated at sub‐1 V regime, displaying clear saturation and fast switching (≈200 µs). The vertical geometry and the use of an electrolyte anticipated that the EG‐VOFET would become a promising candidate for printed logic and drivers with low operating voltages.^[^ ^32^ ^]^ Then, Kawahara et al. raised expectations by reporting the realization of a fast‐switching printed IGVT compatible with plastic and paper substrates.^[^ ^95^ ^]^ The transistors were fabricated along both faces of the paper, and connections were made using photoablation or pin‐driven punch‐through. Electrolyte‐gating and electrochemical modulation of the charge transport in the channel bulk of IGVT allowed Kawahara et al. to control the electrical current flowing between the two faces of the substrate.^[^ ^95^ ^]^ A completely new IGVT architecture was demonstrated by Kim et al. just a few years later,^[^ ^96^ ^]^ elevating expectations once more toward advanced flexible electronics applications and logic gates. They developed an EG‐VOFET based on ionic‐gel dielectrics, metallic heterostructures, graphene, and organic semiconductors. Their EG‐VOFET showed well‐controlled p‐ and n‐type properties in the sub‐1 V regime, leading to current densities >100 mA cm^−2^ and ON–OFF current ratios (*I*_ON/OFF_) >10^3^.^[^ ^96^ ^]^ The EG‐VOFETs were then applied as complementary inverters, NANDs, and NORs, all on plastic substrates. These achievements provided a plethora of possibilities for realizing flexible, transparent, and low‐power neuromorphic bioelectronics in the following years.^[^ ^96^ ^]^

The succeeding insightful view of the IGVT research community was provided by Baby et al. who have fabricated and characterized a printed IGVT that decoupled important device's counterpart sizes from the patterning resolution.^[^ ^97^ ^]^ The IGVT's printed porous semiconducting film, sandwiched by *S* and *D*, provided vertical channels shorter than 50 nm. Such characteristics, along with the use of a polymer‐electrolyte *G*, resulted in a remarkable current density in the ≈1 MA cm^−2^ range. Baby et al. summarized their findings by arguing that, due to the great surface of IGVT's functional parts and the small electronic transport path, the vertical configuration may be appealing for new systems, mainly those where signal amplification and improved multi‐sensing capabilities are required (viz., for ion‐responsive transistors and highly sensitive (bio)sensors.^[^ ^97^ ^]^

The fabrication of vertically stacked ion‐gating devices employing a porous top electrode that allows the electrolyte to come into contact with the OSC channel is a feasible approach for IGVTs.^[^ ^98^ ^]^ Nogueira et al. presented an EG‐VFET based on a low‐cost spray‐deposited zinc oxide film that was electrically connected with silver (Ag)‐nanowire (AgNW) layers, both on an indium tin oxide (ITO) substrate, to form a Schottky contact.^[^ ^99^ ^]^ The findings allowed one to recognize the two fundamental cells of the vertical EG‐VFET, viz. the capacitive‐ and diode cells.^[^ ^99^ ^]^ The evaluation of the capacitive cell showed the current was mainly capacitive, with no major faradaic contribution. By acquiring transfer curves for the EG‐VFETs, Nogueira et al. observed for *V_G_* > 0 an increase in transconductance at *V_D_* < 0, which indicated the diode reverse‐bias switching. The leakage current was significantly lower than the *D* current (*I_D_*) and remained mostly unaffected by *V_G_*. The authors thus pointed out that this behavior confirms that the *I_D_* modulation was a result of the field effect through the AgNW intermediate electrode, facilitated by the electrolyte cations. Their findings provide valuable insights into the production and characterization of low‐voltage EG‐VFETs,^[^ ^100^ ^]^ which are promising systems for the integration of IGVTs within low‐cost printed electronics manufacture, with potential impact on future optoelectronic‐ and neuromorphic applications.

**Figure 3:**
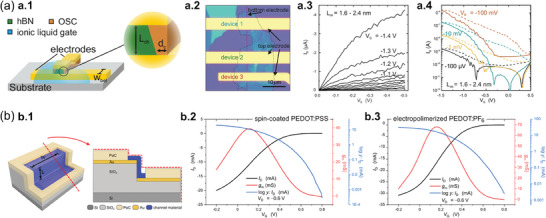
IGVT devices, materials, and electrical characterization. a) EG‐VOFETs with ultrashort *L* values. a.1) Illustration of the device architecture. The zoomed‐in image shows that the spacer thickness is the *L* of the transistor. a.2) Optical microscopy image of EG‐VOFETs with three top terminals sharing the same bottom electrode and the same hBN layer. a.3) Output curves acquired at a *V_D_* sweep rate of 10 mV s^−1^ and a.4) transfer curves measured at a *V_G_* sweep rate of 20 mV s^−1^. In both electrical characteristics, the solid lines are related to the forward sweep direction, and the dashed lines in a.4) correspond to the backward one. Adapted with permission.^[^ ^37^ ^]^ Copyright 2021, American Chemical Society. b) Scalable and reliable method for downsizing *L* of VOECTs. b.1) Sketch of the proposed device composed of a spin‐coated PEDOT:PSS layer together with its cross‐section view. Transfer curves of b.2) a 400 nm‐thick spin‐coated PEDOT:PSS VOECT and b.3) a 280 nm‐thick electropolymerized PEDOT:PF_6_ device. All VOECTs had *W* = 100 µm and *L* = 350 nm. Adapted with permission.^[^ ^94^ ^]^ Copyright 2023, Brodský et al. Published by American Chemical Society.

Lenz et al. demonstrated EG‐VOFETs with ultrashort *L* values down to 2.4 nm (**Figure** **3**a).^[^ ^37^ ^]^ The devices comprised an hBN spacer between the vertically stacked gold (Au) *S* and *D* (Figure 3a.1),^[^ ^101^ ^]^ while the OSC was prepared with poly(diketopyrrolopyrrole‐terthiophene) (PDPP). Figure 3a.2 is an optical microscopy image of a real sample with three top terminals sharing the same bottom electrode and hBN layer. Figure 3a.3 shows the output curves for the ultrashort *L* device with defined saturation regions. In addition, the transfer characteristics in Figure 3a.4 exhibited the OFF current (*I*_OFF_) also increasing as *V_D_* increased, which was due to the large electric field that led to an increment in the leakage current through PDPP or hBN. Even so, the EG‐VOFETs proposed by Lenz et al. exhibited output current densities of 2.95 MA cm^−2^ at *V_D_*  =  −0.4 V, *I*_ON/OFF_ > 10^4^, subthreshold swing (*SS*) of 65 mV dec^−1^, and maximum transconductance (*g*_m_) of 714 S m^−1^.^[^ ^37^ ^]^ Such results highlighted the potential of EG‐VOFETs with ultrashort *L* to be integrated with other devices and for investigating nanoscopic charge transport due to their reduced sizes.

Figure 3b depicts the characteristics of VOECTs developed by Brodský et al. that comprised a spin‐coated poly(3,4‐ethylenedioxythiophene) doped with poly(styrenesulfonate) (PEDOT:PSS) layer or an electropolymerized PEDOT:tetrabutyl‐ammonium‐hexafluorophosphate, (PEDOT:PF_6_).^[^ ^94^ ^]^ The device architecture is illustrated in Figure 3b.1 and is compatible with different OSC deposition processes for the investigation of new materials. Furthermore, the values of channel width (*W*), *L*, and nominal depth (*d*) can be adjusted by changing the thickness of the etched part of the silicon dioxide (SiO_2_) layer. Figure 3b.2 shows the transfer and transconductance curves for the PEDOT:PSS‐based VOECTs with *d*  =  400 nm. Figure 3b.3 exhibits the same electrical curves for PEDOT:PF_6_‐based devices with *d*  =  280 nm. Both devices had *W*  =  100 µm and *L*  =  350 nm. The spin‐coated channel led to high *I*_ON_ ≈ 20 mA, low *I*_OFF_ ≈ 290 nA, and *g*_m_ ≈ 38 mS. The electropolymerized channels exhibited high *I*_ON_ and *g*_m_ in more positive *V_G_* but also had a high *I*_OFF_ ≈ 2 µA and operated at a slower speed than the PEDOT:PSS devices. The findings of Brodský et al. successfully described a simple, scalable, and reliable method to obtain sub‐micrometer *L* for VOECTs, still maintaining good performance of the devices.^[^ ^94^ ^]^

### 3.2 Electronic Characterization of IGVTs

**Figure 4:**
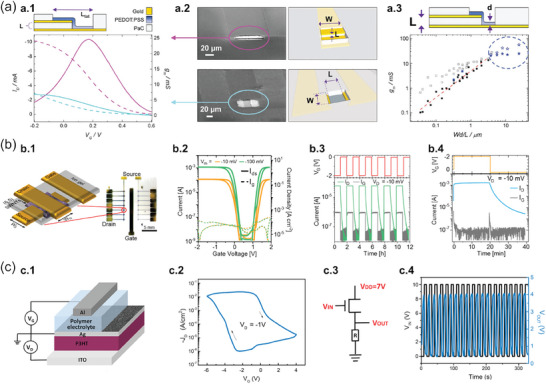
High‐performance IGVTs and the IGVT‐based inverter. a) Influence of transistor geometry on its performance. a.1) VOECT architecture with a PaC spacer between *S* and *D*. The *L* is equal to the PaC thickness. The bottom panel shows the transfer curves (dashed lines) acquired at *V_D_ *= −0.6 V and the transconductance curves (solid lines) for vertical and planar transistors. VOECT responses are shown in magenta, and responses for the planar device are in cyan. a.2) SEM images (left column) and sketches (right column) of the VOECT and planar device. The images illustrate the *L* and *W* properties of the transistors according to their geometries. a.3) The upper panel illustrates *d* of the channel material. The bottom panel exhibits the influence of the *W*, *d*, and *L* properties on *g*_m_. Blue stars are *g*_m_ for VOECTs. The squares correspond to data for planar devices acquired by Donahue et al. (blue squares)^[^ ^66^ ^]^ and by Rivnay et al. (black squares).^[^ ^64^ ^]^ Full symbols designate the experimentally obtained transconductance, whereas the open ones relate to intrinsic transconductance. Adapted with permission.^[^ ^66^ ^]^ Copyright 2017, WILEY‐VCH. b) Vertical transistor geometry for dynamic measurements. b.1) Sketch of the IGVT fabricated with aerosol‐jet‐printed layers of polymer‐sorted (6,5) SWNTs, evaporated Au or printed Ag nanoparticles terminals, and an ionic‐gel electrolyte. The real sample contains 14 transistors and one *G* as shown in the optical microscopy image. b.2) Transfer curve and b.3) switching behavior for IGVTs without metallic SWNTs and pure ionic liquid electrolyte. In b.3), the device was turned on or off by changing *V_G_* from −0.2 V to 0 V, respectively. b.4) Switching behavior for IGVT composed of aerosol jet printed Ag nanoparticle electrodes and (6,5) SWNTs. *V_G_* ranged from 0 V to −2 V, changing after 20 min to 0.5 V. Adapted with permission.^[^ ^55^ ^]^ Copyright 2018, American Chemical Society. (https://pubs.acs.org/doi/full/10.1021/acsanm.8b00756. Further permissions related to the material excerpted should be directed to the ACS.) c) Possibility of using IGVTs in simple circuits. c.1) Illustration of the device structure composed of PEDOT‐coated ITO, P3HT as the channel material, a polymer electrolyte, a 30 nm thick Ag porous electrode, and an Al *G*‐terminal. The P3HT thickness corresponds to *L*. c.2) Transfer characteristic for the IGVT. c.3) Circuit diagram of the inverter based on IGVT loaded as a resistor. c.4) Dynamic responses of the inverter to rectangular voltage input. Adapted with permission.^[^ ^102^ ^]^ Copyright 2018, American Chemical Society.

To assess the performance of the vertical devices appropriately, the influence of device geometry on the materials' electronic features must be evaluated. In 2018, Donahue et al. reported on the influence of vertical geometry on the transistor performance, **Figure** **4**a.^[^ ^66^ ^]^ They evaluated VOECTs with channels composed of ion‐permeable PEDOT:PSS, as exhibited in the upper panel of Figure 4a.1. The vertically stacked Au *S* and *D* terminals were separated by a parylene‐C (PaC) layer. The PaC film thickness was the *L*. The bottom panel of Figure 4a.1 shows the transfer (dashed lines) and transconductance (solid lines) curves for vertical (magenta data) and planar (blue data) transistor channels. The left‐hand column of Figure 4a.2 shows the scanning electron microscopy (SEM) images for vertical and planar devices. The difference between the VOECTs and the planar transistors is in the *L* value as illustrated in Figure 4a.2. For planar devices, *S* and *D* terminals are at the same level and their distance defines *L*. In addition, *g*_m_ depends on the geometric parameters of the transistor channel in a relationship of *Wd/L*, as shown in Figure 4a.3 (top sketch). The bottom panel of Figure 4a.3 shows *g*_m_ values acquired with devices having different *Wd*/*L* ratios. Data represented by squares were for planar devices and by stars for vertical transistors. The blue symbols correspond to the data acquired by Donahue et al. (blue squares),^[^ ^66^ ^]^ while the black symbols are data measured by Rivnay et al.^[^ ^64^ ^]^ The findings of Donahue et al. demonstrated that their VOECTs exhibited a downscaled footprint, an increased intrinsic transconductance (≈57 mS), and a high transconductance normalized by the transistor geometry (814 S m^−1^).^[^ ^66^ ^]^

Another way to obtain vertical channels for transistors is with the deposition of the OSC material between the vertically stacked *S* and *D* terminals. Rother et al. (Figure 4b) employed aerosol jet printing to sandwich the semiconducting layer of polymer‐sorted (6,5) (SWNTs) between evaporated Au or printed Ag nanoparticle terminals.^[^ ^55^ ^]^ Figure 4b.1 illustrates the ion‐gel‐gated vertical device that comprises a 14‐transistor sample with a single *G* terminal. Figure 4b.2 exhibits transfer characteristics acquired for devices gated by a pure ionic liquid of 1‐ethyl‐3‐methyl‐imidazolium‐tris(pentafluoroethyl)‐trifluorophosphate ([EMIM][FAP]) and composed of a metallic nanotube‐free SWNT layer and Au electrodes. The curves led to *I*_ON/OFF_ > 10^4^, *I*_ON_ density > 25 A cm^−2^, and *SS* < 150 mV dec^−1^. The dynamic measurements in Figure 4b.3 show the device switching on in seconds at *V_G_*  =  −2 V and completely switching off in 30 min at *V_G_*  =  0 V. Rother et al. also investigated a [EMIM][FAP]‐ion‐gel‐gated device with the aerosol‐jet‐printed Ag nanoparticle electrodes with a ≈200 nm‐thick‐(6,5) SWNT layer as the semiconducting material.^[^ ^55^ ^]^ The transfer characteristics for these devices showed *I*_OFF_ < 2 µA at *V_D_*  =  −10 mV, and *I*_ON/OFF_ > 10^3^. The dynamic cycles in Figure 4b.4 exhibit the devices turning on within seconds and turning off within a longer time. This behavior was due to the low *V_G_* leading to a slow ion movement and a small electric field.

Another intriguing study on SWNT‐based IGVTs was conducted by Ueji et al.^[^ ^103^ ^]^ This study was pioneering in the field as they developed a method to evaluate the materials' electrical and thermal characteristics in a single device platform (EG‐VFET), in the same direction.^[^ ^103^ ^]^ Among their outcomes, they argued that comprehending the correlation between the electrical and thermal properties of thin films is critical to thermal control. They showed that the SWNT film's thermal conductivity is not affected by the high current modulation, over 4 magnitude orders^[^ ^103^ ^]^ For CNT‐based devices, optimizing doping can enhance the power factor without increasing thermal conductivity, thereby improving energy conversion efficiency. Therefore, their findings provided a reasonable approach to improving thermoelectric conversion efficiencies in IGVTs.

Luan et al. demonstrated the use of porous top electrodes to integrate IGVTs in simple circuits (Figure 4c).^[^ ^102^ ^]^ The vertically‐stacked devices ‐ consisting of poly (3‐hexylthiophene) (P3HT) as the channel material, Ag porous *S*, ITO *D*, and aluminum (Al) *G* (Figure 4c.1) ‐ exhibited *I*_ON_ and *I*_OFF_ densities of 10 mA cm^−2^ and 1 µA cm^−2^, respectively, and *I*_ON/OFF_  =  10^4^ (Figure 4c.2). The applied *V_G_* slowly moved the ions through the pores of the *S* to electrochemically stabilize the p‐doped channel, which led to hysteresis in the transfer curves that indicated slow response and long retention time for the transistors. The applicability of the transistors in simple circuits was demonstrated by connecting them in series with a 10 kΩ resistor (Figure 4c.3), which led to an inverter loaded with a resistor having a voltage gain of 7.4. Figure 4c.4 shows 20 cycles of the responses over time for the inverter. The cycles were acquired by applying a rectangular input voltage (*V*_IN_) of zero and 10 V with a 15 s pulse, and the output voltage (*V*_OUT_) of the inverter successfully followed the input signal and reversely switched from 4.0 to 0.5 V. In summary, the findings show devices with simple manufacturing processes and low‐cost material composition. Such characteristics make the devices attractive for the industry to integrate ion‐gating circuits with stacked components.

### 3.3 IGVT‐Based Logic Circuits

**Figure 5:**
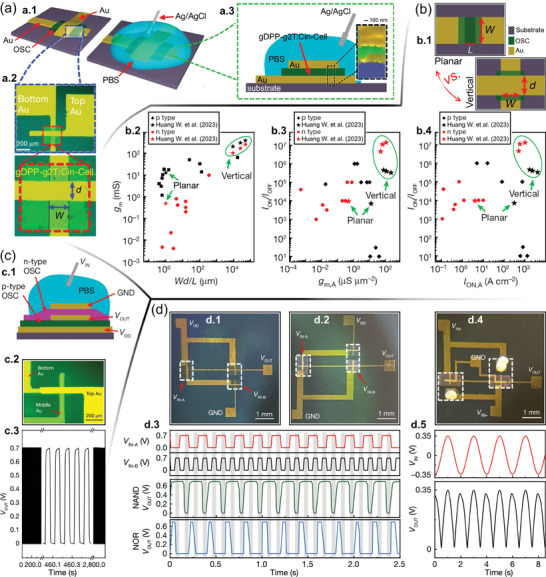
Vertically stacked complementary circuits based on VOECTs. a) Device fabrication, structure, and morphology. a.1) *S*‐OSC‐*D* base structure (left) and assembled VOECT (right‐hand side). a.2) Optical microscopy image of a p‐type VOECT. The electrode overlap region is zoomed in (*W* = *L* = 70 µm). a.3) Cross‐section illustration of p‐type VOECT (zoom‐in: false‐color cross‐section SEM image). b) VOECT performance and comparison with the planar counterpart and the literature. b.1) Schematics illustrating the differences in typical planar‐ and vertical device geometry. b.2–4) *g*_m_ *vs. Wd*/*L*, *I*_ON/OFF_ *vs. g*_m_, and *I*_ON/OFF_ *vs. I*_ON_, respectively, for the reported OECTs.^[^ ^18^, ^40^, ^107^, ^108^, ^110^, ^111^, ^112^, ^113^, ^114^, ^115^, ^116^, ^117^, ^118^, ^119^, ^120^ ^]^ c) Complementary inverter based on 2 VOECTs: c.1) illustration of vertical stacking, c.2) top view of 2‐VOECT inverter, and c.3) 10 Hz switching stability of 2‐VOECT inverter. d) VOECT‐based logic circuits: optical images of d.1) NAND‐ and d.2) NOR circuits, d.3) NAND and NOR input/output characteristics, d.4) optical image of the rectifier, and d.5) rectifier input/output characteristics. Adapted with permission.^[^ ^54^ ^]^ Copyright 2023, Huang et al. Published by Springer Nature.

The application of IGTs along with their accompanying circuitry, using organic‐electrochemical and electrolyte‐gated organic field‐effect transistors (OECTs and EG‐OFET, respectively), shows great potential in the areas of bio‐, wearable, and neuromorphic electronics. This is due to their features related to biocompatibility, operating voltages lower than 1 V, transconductance >10 mS, and power consumption lower than 1 µW.^[^ ^11^, ^22^, ^104^, ^105^, ^106^ ^]^ However, the realization of critical complementary logic OECTs and EG‐OFETs ‐, i.e., the fundamental step toward complex integrated neuromorphic architectures ‐ is currently limited by working and dynamic instability, slow switching, unsuitability for large‐scale integration, and typically poorer n‐type OSC channel performance.^[^ ^47^, ^107^, ^108^, ^109^ ^]^ Motivated by such a challenging scenario, Huang et al. have successfully engineered p‐ and n‐type VOECTs with exceptional performance by combining a redox‐inactive photo patternable polymer with redox‐active ones to create the ion‐permeable OSC duly integrated in a scalable vertical architecture.^[^ ^54^ ^]^ **Figure** **5**a.1 exhibits the schematic illustrations of the device *S*‐OSC‐*D* vertical structure (left) and the assembled VOECT (right‐hand side). The bottom‐ and top electrode widths outline the OSC's *W* and *d*. The zoom in the *S*‐OSC‐*D* is exhibited in Figure 5a.2 by optical microscopy images, whereas the VOECT cross‐section view is sketched in Figure 5a.3. The zoom in Figure 5a.3 exhibits an artificial‐colored SEM image of the cross‐sectional view of the OSC placed between *S* and *D*, whereas *L* is the semiconductor layer thickness, ≈100 nm. Similar‐footprint planar OECTs were fabricated and characterized to evaluate the improvements provided by the vertical geometry. It is worth noting that the VOECT structures can be designed to display smaller footprints since the electrical connection lines additionally function as *S* and *D* contacts. A schematic illustration of both planar‐ and vertical device geometries is presented in Figure 5b.1, whereas Figure 5b.2–4 exhibits a thorough comparison of the planar and vertical device properties with the reported in the literature.^[^ ^18^, ^40^, ^54^, ^107^, ^108^, ^110^, ^111^, ^112^, ^113^, ^114^, ^115^, ^116^, ^117^, ^118^, ^119^, ^120^ ^]^ According to Huang et al.,^[^ ^54^ ^]^ their p‐type VOECTs achieved, per unit area, the highest *g*_m_ and *I*_ON_ compared to all previous reports (Figure 5b.2–4).^[^ ^18^, ^40^, ^107^, ^108^, ^110^, ^111^, ^112^, ^113^, ^114^, ^115^, ^116^, ^117^, ^118^, ^119^, ^120^ ^]^

Huang et al. also evaluated the stability of cycling and transient response of the high‐performance VOECTs.^[^ ^54^ ^]^ Both n‐ and p‐type VOECTs stably achieved >50 000 switching cycles, which is significantly higher than the literature values for OECTs, particularly for n‐type devices.^[^ ^111^, ^121^ ^]^ Due to the distinctive operating mechanism, uncomplicated fabrication methods, and good cycling stability of the VOECTs, the practical realization of vertically stacked complementary inverters was achieved in 2023.^[^ ^54^ ^]^ In the diagram presented in Figure 5c.1, the inverter design is depicted with the n‐type VOECT positioned directly above the p‐type one. The three‐dimensional design allows for significantly higher integration densities, leading to a 50% reduction in footprint per inverter as the optical microscopy image shows in Figure 5c.2. The 2‐VOECT inverter has efficient voltage transition and a maximum gain of ≈150,^[^ ^54^ ^]^ besides displaying outstanding stability for over ≈30 000 cycles (Figure 5c.3). Furthermore, circuitry elements including NAND and NOR logic gates working between 0 V to +0.7 V (Figure 5d.1–3), plus a VOECT‐based rectifier (0.35 V amplitude, Figure 5d.4,5), were successfully fabricated, showcasing the versatility of the VOECT technology. It is worth mentioning that previous NAND and NOR logic gates were made with unipolar p‐type planar OECTs,^[^ ^122^, ^123^, ^124^, ^125^ ^]^ while complementary circuits were restricted to the initial stage of an inverter mainly due to the poor achievements of the n‐type planar OECTs.^[^ ^110^, ^126^ ^]^ The verdict, therefore, is that OECTs not only enable vertically stacked complementary inverters but still simplify the incorporation of electrochemical technologies into more complicated neuromorphic electronics.

**Figure 6:**
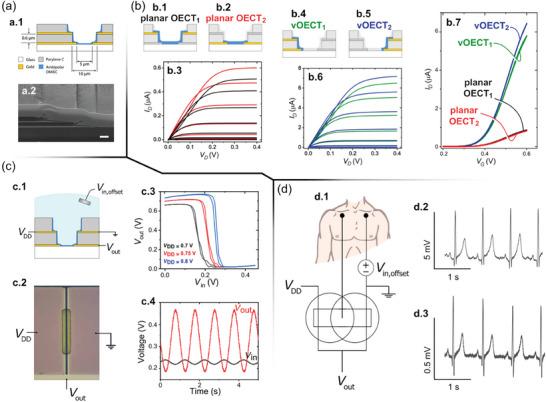
VOECT‐based ambipolar inverters and application as a bio‐signal amplifier. a) Device structure: a.1) cross‐sectional view of the cofacial pair of OECTs illustrating materials, dimensions, and contacts; a.2) SEM image of the cross‐section of the cofacial pair of OECTs. Scale bar, 1 µm. b) Planar‐ and vertical‐device electric characteristics. Panels b.1) and b.2) exhibit the planar OECT configurations, whereas b.3) exhibits their output characteristics. Panels b.4) and b.5) exhibit the VOECT configurations, whereas b.6) exhibits their highly modulated output characteristics (compared with planar OECTs). Panel b.7) depicts the transfer characteristics of the planar‐ and VOECTs. c) VOECTs applied as complementary inverter: c.1) illustration of the cross‐section view of cofacial OECTs circuited as a complementary inverter; c.2) microscopic image of the inverter top view; c.3) transfer curves for the inverter; c.4) sinusoidal input with the corresponding amplified output. d) ECG signal amplification acquired with a complementary inverter. d.1) Voltage preamplifier wiring diagram. Medical electrodes are attached to the right and left sides of the body, below the clavicle. They are connected to a direct current offset and the input of the inverter on a digital multimeter. d.2) Signal recorded from the output of the VOECT inverter. d.3) Signal between the medical electrodes acquired with the digital multimeter. Adapted with permission.^[^ ^131^ ^]^ Copyright 2021, Rashid et al. Published by AAAS.

In bioelectronics, there has been a conventional separation of the initial signal transduction and the subsequent signal processing for recording.^[^ ^127^, ^128^ ^]^ Nonetheless, recent research shows that combining these components to reduce the physical distance between them can lead to better signal quality, and more straightforward implementation, paving the way for practical wireless systems.^[^ ^129^, ^130^ ^]^ This method boasts an array of advantages and deserves careful consideration, especially because the VECTs emerged as pivotal players and potential game‐changers. A recent study by Rashid et al. presented a new active sensing node that utilizes VOECTs to create an ambipolar complementary inverter.^[^ ^131^ ^]^ The inverter was made up of two VOECTs situated on opposite sides of a single active area (viz. cofacial configuration), allowing for a footprint that is the same as a single planar OECT. To create a complementary inverter, two VOECTs were patterned along opposite side walls of a single active area (**Figure** **6**a). This involved photolithography to create the cofacial pair of VOECTs, with metallic layers serving as the *S* and *D* contacts and interconnects. The VOECT *L* was defined by the thickness of the PaC layer, which was ≈600 nm. The VOECTs' *W* and the spacing between VOECTs were defined by the etched area (Figure 6a.1). The SEM image shows the VOECT's layer stacking (Figure 6a.2). The VOECT individual operation was then compared to microfabricated planar OECTs.^[^ ^131^ ^]^ It is worth pointing out that an ambipolar organic mixed ionic‐electronic conductor (OMIEC), i.e., capable of realizing both hole and electron transport under electrochemical modulation in the presence of an electrolyte, was employed as active material. The versatile device architecture allowed the electrical characterization of at least 4 different OECTs, viz. planar OECT_1_ (Figure 6b.1), planar OECT_2_ (Figure 6b.2), VOECT_1_ (Figure 6b.4), and VOECT_2_ (Figure 6b.5). Their n‐channel output characteristics, without generality loss, are exhibited in Figure 6b.3 and Figure 6b.6 for OECTs and VOECTs, respectively. The VOECT architectures provided a tenfold *I_D_* compared to planar OECTs, which agreed with the geometric scaling of the OECT channels (viz. ≈600 nm for VOECTs versus ≈5–6 µm for planar OECTs). The outstanding features of the vertical transistors can also be confirmed by the OECT transfer characteristics in Figure 6b.7. Furthermore, Figure 6b additionally evidences the miniaturization‐ and integration potential displayed by the vertical device architecture, since the VOECT achieved a considerably superior performance along with enabling two devices in the same footprint required by a single lower‐performance planar OECT.

To create a complementary inverter, two different switching devices, namely p‐ and n‐type transistors, are usually wired separately. The p‐type OECT's *S* is connected to the *D* power voltage (*V_DD_*), while the n‐type OECT's *S* is connected to the ground. Both OECTs' *G* terminals are joined externally to create a common input. Finally, *V*_OUT_ is read by connecting the *D* terminals of both OECTs externally. Rashid et al. successfully achieved an inverter configuration that fitted within the same space as a single planar OECT.^[^ ^131^ ^]^ They used a pair of VOECTs in a cofacial configuration, as seen in Figure 6c.1. This configuration shared the electrolyte and *G*, which connected both channels and coupled the input of the two OECTs. Depending on the *V_G_*, one VOECT acted as a p‐type, while the other behaved as an n‐type. The p‐type VOECT's *S* terminal was connected to *V_DD_*, while the n‐type VOECT's *S* terminal was connected to the ground. The bottom contacts of each VOECT were electrically connected and acted as the *D* electrodes that acquired the inverter output signal (*V*_OUT_). Figure 6c.2 shows the cofacial pair optical microscopy image, along with the schematics for inverter measurement.

The inverter voltage transfer characteristics were recorded with varying *V_DD_* (Figure 6c.3), whereas the gain reached a peak of ≈28.^[^ ^131^ ^]^ The alternating current characteristics of the inverter, which revealed a ≈16 Hz cutoff frequency, are presented in Figure 6c.4 (a 10 mV input sine wave with 0.23 V offset at a 1.5 Hz frequency).^[^ ^131^ ^]^ The effectiveness of the VOECT‐based inverter in amplifying bio‐signals is demonstrated through the recording of electrocardiogram (ECG) signals (as shown in Figure 6d). To achieve this, one medical electrode was wired to the inverter input (*G*), while the other was wired to a voltage source configured for an offset bias in which peak gain happened (Figure 6d.1). The ECG response from the inverter had a 5 mV‐peak‐to‐peak amplitude (Figure 6d.2), while the response acquired between the two medical electrodes using a digital multimeter led to a 0.5 mV‐peak‐to‐peak amplitude (Figure 6d.3). The measured gain, ≈10,^[^ ^131^ ^]^ agreed with previously observed alternating current measurements (Figure 6c.4).

**Figure 7:**
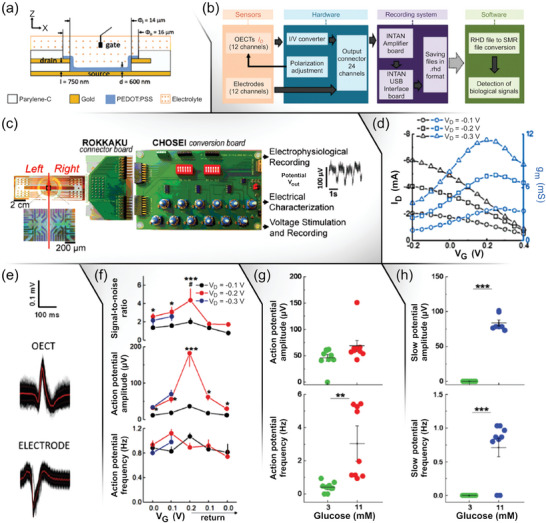
VOECTs for low‐amplitude micro‐organ signal processing. a) VOECT geometrical structure. b) Block diagram of the data flow and acquisition process. c) Device‐specific board connecting all VOECTs/electrodes to the CHOSEI board. d) *I_D_ vs. V_G_* and *g*_m_ *vs. V_G_* curves of VOECT array covered with HL‐1 cells for the recording of action potentials. e) Extracted mean configuration of HL‐1 action potentials obtained using VOECTs in comparison with electrodes. f) Signal‐to‐noise ratios, action potential amplitudes, and action potential frequency of HL‐1 cells. g,h) Action potential amplitudes and frequencies, and slow potential amplitudes and frequencies at low and high glucose concentrations. Adapted with permission.^[^ ^138^ ^]^ Copyright 2022, Abarkan et al. Published by Wiley‐VCH.

The noteworthy characteristics of OECTs, such as biocompatibility and form factor, have been widely used in brain and heart recording applications,^[^ ^132^ ^]^ as well as in cellular recordings of cardiomyocytes.^[^ ^29^, ^133^, ^134^ ^]^ Nevertheless, some important problems, such as operational stability (important for obtaining long‐term quantitative data of living matter), still need to be addressed. Similarly, the preparation of multichannel OECTs needs fine probe control, which can be achieved by calibrating the corresponding electrical circuits to obtain the relevant long‐term quantitative readouts. It must also be considered that electrogenic cells exhibit responses with distinct amplitudes. Cardiomyocytes or neurons depolarize to significantly more intense values (+40 mV) compared to cells of the endocrine system, such as pancreatic islets (0 mV), necessary for nutrient balance.^[^ ^135^, ^136^, ^137^ ^]^ While keeping in view these considerations, Abarkan et al. demonstrated the ability of OECTs to be used with micro‐organs, such as the islets that are fundamentally harder to monitor.^[^ ^138^ ^]^ The devices were fabricated in the vertical architecture (viz. VOECTs; **Figure** **7**a), which assisted in increasing the spatial resolution since a higher number of transistors (12) could be arranged in a given geometrical area. To provide voltage bias, connect sensor devices, and convert *I_D_* into readable voltage signals, the authors created a custom circuit board (ROKKAKU), including individually tunable *V_D_* to gain homogeneity (Figure 7b). To record all the signals simultaneously, the authors also made sure that the positions of the OECTs and electrodes on the sensor matched the connection board (Figure 7c). Subsequently, a conversion and polarization board (CHOSEI) was used to convert OECT currents to voltages via a 560 Ω load resistor, and a 24‐pin output connector was used to connect a recording system (INTAN) to the OECTs and electrodes. To assess the utilization of VOECTs for micro‐organ or cell recordings, the electrical characteristics were measured in a culture medium containing serum, potassium chloride solution, and physiological buffered salt solution, and the devices showed stable behavior for up to 10 days. The stability of the biological preparation on the array was evaluated by using cardiac cells and measuring the electrical behavior after 6 days of culture on VOECTs. The resulting *I_D_*/*V_G_* and *g*_m_/*V_G_* curves of VOECTs covered with cardiac muscle‐derived HL‐1 cells exhibited a maximum of 0.2 V, as shown in Figure 7d.

The reliable detection of action potentials, guaranteed by choosing a 100 Hz low‐pass filter and a 10 Hz high‐pass filter, was found to be robust over a large range of the adaptive threshold.^[^ ^138^ ^]^ Abarkan et al. were also successful in evaluating the frequency of cardiomyocyte action potentials from the interval between the peaks by describing a normal distribution.^[^ ^138^ ^]^ At *V_D_*  =  *V_G_*  =  0.2 V, the mean shape of action potentials represented the exact inverse of the action potentials recorded by the electrodes manufactured on the chip that contained the VOECTs (Figure 7e). On the other hand, the measurement of the electrophysiological signals revealed a signal‐to‐noise ratio of 3–6, while the frequency of action potentials was found to be unchanged during different electrical conditions (Figure 7f). The signal amplitude was found to be primarily affected by the cell/sensor resistance and the cell coverage, and the stable responses for action potential frequency and shape showed the independence of the cells' biological operations to the electrical parameters.^[^ ^138^ ^]^ After establishing the stable *V_D_* and *V_G_* regions and validating the electronic board, the authors also demonstrated the recording of pancreatic islets, and after exposing the devices to glucose it was found that a change in the voltages modified *g*_m_ but not the behavior of the cells. In addition, action potentials and slow potentials were found to be glucose‐dependent, reflecting nutrient‐induced islet activation (Figure 7g,h).

The work of Abarkan et al. aimed at providing physiologically quantitative recordings using VOECT devices by choosing polarization procedures in biological circumstances and demonstrating in pancreatic islets the extraction and recording of cellular events and a micro‐organ.^[^ ^138^ ^]^ One of the problems faced by the authors corresponds to the reduction of the performance of VOECTs when an additional resistive layer was introduced and the devices were exposed to a culture of cells. The problem was found to persist even after cell removal, perhaps because of the shedding of extracellular matrices. It is important to highlight that many studies related to the use of planar OECT in cellular research have overlooked this concern, and there remains ambiguity as to whether the reported data pertains to the extent/duration of prior polarization or to device characterizations conducted in the absence/presence of cells. Therefore, more work focusing on carefully controlling the device parameters and obtaining quantitatively reliable data is required. Similarly, obtaining a homogeneous bias is also critical for biological recordings since small variations can lead to different signal amplifications, which in turn results in incorrect determination of frequencies. Another important aspect of obtaining reliable data involves the reliable interfacing of organic materials and biological substrates. This was demonstrated by the authors when they applied different biases and did not observe changes in signal propagation or frequency of biological signals in islet cells or cardiomyocytes.^[^ ^138^ ^]^ In summary, Abarkan et al. demonstrated the ability of VOECTs to capture both slow and rapid signals and showed that both cells/tissues (with high amplitude signals), as well as electrogenic cells (with low amplitude signals), are accessible to OECTs.^[^ ^138^ ^]^

When discussing logic circuits, the possibility of using gel electrolytes is important, as they offer an advantage over liquid electrolytes, provided that the latter can affect neighboring devices and create inappropriate electrochemical connections or artifacts. In a recent study, Jeong et al. successfully demonstrated a quasi‐solid‐state ion gel‐gated VOECT using a quasi‐solid electrolyte.^[^ ^139^ ^]^ Compared to traditional aqueous devices, the VOECTs in this study showed an enlarged electrochemical window, which means they can withstand higher voltages and improved operational stability. Notable advantages of the VOECTs used in this study were the large area (50 × 50 µm) and short *L* (≈30 nm) of the channel. Overall, the authors demonstrated a significantly increased *g*_m_ of 72.8 mS and the use of p‐type and n‐type VOECTs to create logic gates. In another report, Koutsouras et al. described a method that involved the use of electrodeposition to fabricate integrated VOECTs with *L* as short as 60 nm, resulting in very high transconductance of up to 275 mS.^[^ ^140^ ^]^ Such stimulating results, and the potential of VOECTs to provide high amplification and small footprint, strongly indicate the potential of these devices in next‐generation bioelectronic applications, such as neuromorphic devices, organ‐on‐a‐chip technologies, and implantable systems.

The emergence of OECTs has also opened up new possibilities for printable electronics technology. However, these devices have been limited by their low operation speed and bulky device geometry, which hinder their integration into high‐functioning logic circuitry. To overcome these limitations, multivalued logic transistors (MVT) are gaining prominence recently.^[^ ^141^ ^]^ MVTs are advantageous due to data processing, circuit design, and power efficiency.^[^ ^142^, ^143^ ^]^ By utilizing the multivalued logic architecture in OECTs, the integration level for logic operations can be significantly enhanced, which is not achievable with conventional binary logic OECTs. Additionally, the chemical versatility of organic materials allows for finely optimized multistate electrical states, meeting the required figure of merits for MVTs. Therefore, by developing strategies to realize OECT‐based MVTs, the operation frequency can be improved and a broader range of materials can be utilized. This expands the applicability of OECTs and establishes devices with diverse material options from the perspective of MVTs.

**Figure 8:**
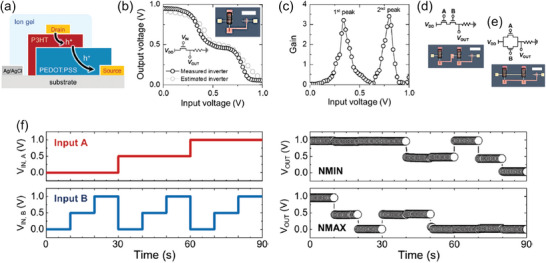
Monolithic tandem VOECTs for printed multivalued logic applications. a) Device geometry of dual‐channel VOECT with P3HT‐PEDOT:PSS vertically stacked channels. b) Simulated and measured *V*_OUT_ *vs. V*_IN_, and c) signal gain of a ternary inverter based on a dual‐channel VOECT. d) Logic circuit diagrams and images of the VOECT‐based NMIN gate and the e) VOECT‐based NMAX gate. f) *V*_OUT_ and *V*_IN_ as a function of time of NMIN and NMAX gates. Adapted with permission.^[^ ^31^ ^]^ Copyright 2023, Wiley‐VCH.

In recent work, Lim et al. presented a method for tuning multistate VOECTs by chemical modifications and for designing fast‐response printing multi‐valued logic circuits.^[^ ^31^ ^]^ The approach consisted of using a monolithically stacked heterogeneous dual‐channel architecture by consecutively depositing two widely used electrochemical materials, PEDOT:PSS and P3HT (**Figure** **8**a). These layers were then penetrated and doped by electrolyte ions gradually by voltage application, which assisted in inducing an onset behavior in the electrical conductivity, resulting in multiple distinctive and stable logic states. Additionally, the authors demonstrated that the *G* driving voltage of the VOECTs could be easily modulated by moderate chemical modifications on PEDOT:PSS, which allowed for electrical optimization. Using the optimized dual‐channel VOECTs, it was possible to design and fabricate an array of printable ternary logic circuits, such as a NOT gate and inverter, with fast switching and drive voltage < 1 V. In Figure 8b, a ternary inverter composed of dual‐channel VOECT is shown. An electrochemical potential (*V_G_* plus *V_D_*) is the turn‐on voltage of the dual‐channel VOECT in the inverter, unlike that applied to a single transistor.^[^ ^31^ ^]^ As a result, the current‐drop‐point, or the first turn‐on voltage, occurred earlier in the inverter compared to a single transistor.^[^ ^31^ ^]^ This led to the occurrence of an intermediate state at a *V_G_* below 0.45 V (Figure 8b).

In the application shown in Figure 8, the inverter demonstrated quasi‐symmetric *V*_IN_‐*V*_OUT_ transfer characteristics at a drive voltage of <1 V.^[^ ^31^ ^]^ These achievements of the symmetric inverter are suitable for applications in more complex logic circuits, such as XOR or LATCH, where the *V*_OUT_ is reused as a *V*_IN_. Figure 8c shows the gain values of the multistate inverter at a *V_DD_* (*D*‐to‐*S* voltage) of ≈1 V. The gain was almost zero in the region between the two peaks, indicating a rational flat intermediate state. The inverter stability was assessed by analyzing the static noise margin in a symmetric *V*_IN_‐*V*_OUT_ transfer curve. Furthermore, the dynamic switching property of the ternary inverter was demonstrated, showing its ability to realize continuous ternary logic circuits. Finally, the authors fabricated NMIN and NMAX logic gates, which exhibited similar functions to NAND and NOR logic gates, respectively.^[^ ^31^ ^]^ Figure 8d,e shows the circuit diagrams and optical microscopy images for the NMIN and NMAX gates. The dynamic measurements for the NMIN and NMAX gates are shown in Figure 8f. In summary, this work is a great example showing the potential of VOECTs in bioelectronics, wearable electronics, and artificial neuromorphic electronics. In particular, VOECT‐based logic circuits can provide the building blocks for neuromorphic systems, enabling the development of energy‐efficient, adaptive, and intelligent computing platforms that can mimic the behavior of biological neural networks.

### 3.4 IGVT‐based Multi‐Parametric Applications

**Figure 9:**
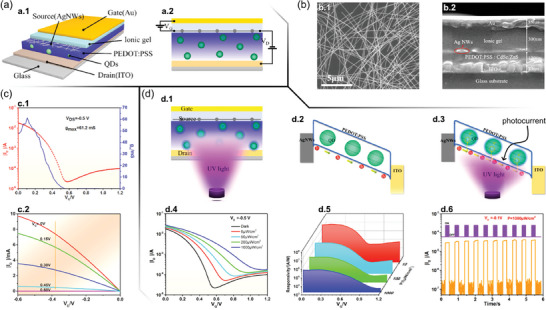
Vertical‐channel organic/inorganic hybrid electrochemical phototransistors. a) Schematic illustrations of a.1) the hybrid optoelectronic device based on the VECT architecture with nanoscale *L*, and a.2) the VECT electrical circuit. b) SEM images of b.1) the AgNW *S*, and b.2) the VECT cross‐section. c) Electrical characterization of the VECT: c.1) transfer‐ and transconductance characteristics, and c.2) output characteristics. d) Phototransistor working principle and optoelectronic performance: d.1) illustration of the phototransistor under 365 nm illumination, d.2) schematic mechanism of VECT conduction, d.3) schematic mechanism of the UV light‐induced photocurrent, d.4) transfer curves acquired at dark condition and different UV intensities, d.5) responsiveness for *V_G_* under various illumination intensities, and d.6) temporal response to 10 periods of 365 nm illumination with 1000 µW cm^−2^. Adapted with permission.^[^ ^148^ ^]^ Copyright 2021, American Chemical Society.

With the latent possibility of enhancing energy efficiency and boosting the performing speed of neuromorphic computing technologies, optoelectronic neuromorphic systems are nowadays enthusiastically investigated.^[^ ^144^ ^]^ Considering the electrical neuromorphic devices, the speed of computation may often be restricted by the trade‐off between bandwidth, connection, and density.^[^ ^145^ ^]^ In contrast, optoelectronic neuromorphic devices can use light as the stimulation source to achieve superior speeds and bandwidths, reduced crosstalk, and more efficient power consumption.^[^ ^146^, ^147^ ^]^ It is also noticeable that the photonic‐based neuromorphic architectures can withstand up to 20% of device faults,^[^ ^144^ ^]^ which represents a sympathetic level of tolerance that the conventional architectures are not capable of reaching.^[^ ^144^ ^]^ Although no in‐depth investigations of EG‐VFET and VECT‐based neuromorphic applications have been reported so far, in 2021 Yan et al. reported their exciting achievements on the so‐called vertical‐channel organic/inorganic hybrid electrochemical phototransistor.^[^ ^148^ ^]^ Such a timely contribution is undoubtedly the first checkpoint on the road to the practical realization of EG‐VFET and VECT‐based neuromorphic devices. **Figure** **9**a.1 exhibits the schematic of the inorganic/organic hybrid IGVT with a nanometric channel (*L*  =  120 nm), whereas Figure 9a.2 illustrates the device's cross‐sectional view along with the electrical circuit scheme. To enable *G* to control the transistor operation through the ion migration toward and within the transistor channel, AgNWs were employed as the porous *S*, as shown by the SEM image in Figure 9b.1. Figure 9b.2 exhibits the cross‐sectional view of the proposed phototransistor, evidencing its different layers (viz. ITO, cadmium selenide (CdSe)/zinc sulfide (ZnS)‐PEDOT:PSS, AgNWs, ionic gel, and Au).^[^ ^148^ ^]^

Figure 9c.1 exhibits the VECT's transfer and transconductance characteristic curves.^[^ ^148^ ^]^ As *I_D_* decreases with increasing *V_G_*, the device is a depletion‐type electrochemical transistor. Figure 9c.2 shows the output characteristic curve, which shows an increase in *I_D_* with *V_D_* without reaching saturation.^[^ ^148^ ^]^ In principle, the devices operate in the ON state at zero gate voltage due to the intrinsically high conductivity of the active layer.^[^ ^149^ ^]^ Application of a positive *V_G_* causes lithium cations (Li^+^) to enter the channel via the perforations of the AgNW electrode, compensating for sulfonate groups on the PSS. Meanwhile, the bis(trifluoromethanesulfonyl)imide anions (TFSI^–^) are transported toward *G*, creating an EDL between the ionic gel and the *G* terminal. This process leads to the dedoping of PEDOT:PSS, reducing the hole current.^[^ ^149^ ^]^ The positive *V_G_* increases the energy barrier width at the PEDOT:PSS/AgNW interface, possibly because of the resulting decrease in the potential energy of PEDOT:PSS,^[^ ^150^, ^151^, ^152^ ^]^ which acts as a hindrance for hole injection from *S* to the device channel. Eventually, the combined effect of PEDOT:PSS dedoping and the increased barrier width brought about by the positive *V_G_* assist the devices in turning OFF.

Succeeding the electrical characterization, Yan et al. proceeded to demonstrate the phototransistor application under 365 nm, which agreed with the absorption spectrum of the VECT's CdSe/ZnS quantum dots (QDs),^[^ ^148^ ^]^ as illustrated in Figure 9d.1. The authors defended that their phototransistor working principle (Figure 9d.2,3) was based on excitons (i.e., electron‐hole pairs), which were produced in the CdSe/ZnS QDs when device was illuminated by UV light. Due to the energy band matching, photogenerated holes shifted from CdSe/ZnS QDs to PEDOT:PSS,^[^ ^148^ ^]^ leaving photogenerated electrons in the conduction band of the QDs (Figure 9d.3). As a result, the device transfer characteristics obtained as a function of the UV illumination (Figure 9d.4) showed a significant increment in *I_D_* when the devices were illuminated.^[^ ^148^ ^]^ As shown in Figure 9d.5, the responsivity could be controlled by *V_G_* and UV‐light intensity, whereas the latter decreased when the illumination intensity increased due to the higher level of photocarrier recombination caused by the UV light.

In accordance with the aforementioned, the demonstrations in Figure 9 showcase appealing neuromorphic features and hold great promise.^[^ ^148^ ^]^ Figure 9d.6 displays the photoresponsive behavior of the devices when the light is turned on and off. The vertical phototransistors provided a consistent and reversible *I_D_* value for different light intensities.^[^ ^148^ ^]^ Once the device was illuminated, the photocurrent quickly reached its maximum value and stabilized for a period. When the light source was switched off, the current dropped to its initial value. The calculated on‐ and off response times were 73 and 123 µs, respectively.^[^ ^148^ ^]^ Such response times were significantly faster than those of planar‐architecture electrochemical phototransistors and the majority of field‐effect transistor photo‐switches.^[^ ^21^, ^153^, ^154^, ^155^, ^156^ ^]^ Given all the outstanding properties and the unique dynamic response reported for the vertical electrochemical phototransistor, we argue that reliably assessing the neuromorphic optoelectronic functions of VECTs and EG‐VFETs is an important ongoing challenge. We foresee that VECTs and EC‐VFETs, along with their integrated optoelectronic circuits, are promising candidates for showcasing cutting‐edge demonstrations of various synaptic and neuromorphic multi‐parametric functions. These functions include the system's photo‐dependent properties such as STP and LTP, STDP, PPF, and PPD. We firmly believe that these demonstrations will establish new paradigms in neuromorphic device research in the next years.

**Figure 10:**
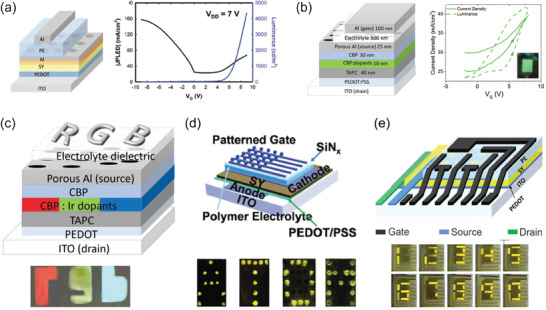
Light‐emitting EG‐VOFETs. Structures of light‐emitting EG‐VOFETs where the porous *S* of Al enables contact between the electrolyte and the organic layer comprised of: a) the SY polymer emitter, and b) a mixture of CBP host doped with Ir‐dopant phosphorescent guest. The electrical characteristic curves for the devices are exhibited in the right‐hand panels in (a,b). c) Light‐emitting EG‐VOFET‐based prototype display composed of a light‐emitting material doped with three different‐color components. The three *G* electrodes were patterned to display in red, green, and blue the initial letters of each color. d) An array consisting of *G* terminals that can be individually turned on to display letters. e) Light‐emitting EG‐VOFET with seven *G* electrodes that are controlled at different times to display numbers from 0 to 9. (a,d) Adapted with permission.^[^ ^159^ ^]^ Copyright 2016, WILEY‐VCH. (b,c) Adapted with permission.^[^ ^160^ ^]^ Copyright 2017, American Chemical Society. e) Adapted with permission.^[^ ^161^ ^]^ Copyright 2017, American Chemical Society.

Vertical light‐emitting transistors are generally composed of a capacitive component stacked on top of a light‐emitting element layer and a porous electrode that allows the electric field or ECD process generated by *G* to reach the light‐emitting component to modify the injection or transport of charges.^[^ ^157^, ^158^ ^]^ Luan et al. developed the first light‐emitting EG‐VOFET in 2016 (**Figure** **10**a).^[^ ^159^ ^]^ The device consisted of a stacked light‐emitting bottom composed of ITO, PEDOT, super yellow (SY) polymer emitter, and Al layers. At the top, the device was composed of an Al *G* terminal deposited on a polymeric electrolyte made of poly(ethylene oxide) (PEO), lithium triflate (LiCF_3_SO_3_), and poly(methyl methacrylate) (PMMA). The light‐emitting EG‐VOFET transfer characteristics showed the device switching on by applying *V_G_*  =  8 V (right panel of Figure 10a). The highest value for luminance was 4500 cd m^−2^, and the external quantum efficiency was 1.7%, which indicated the device's capability in signage displays.^[^ ^159^ ^]^

The light‐emitting EG‐VOFET architecture allows the change of the porous electrode material for a compound of Al and lithium fluoride (LiF) to improve the electrochemical performance,^[^ ^161^ ^]^ or even the replacement of the light‐emitting polymer.^[^ ^160^, ^162^ ^]^ Liu et al., for instance, developed a light‐emitting stack with ITO, PEDOT, 4,40‐cyclohexylidenebis[*N,N*‐bis(4‐methylphenyl)benzenamine] (TAPC), 4,4‐bis(N‐carbazolyl)−1,1‐biphenyl (CBP) with iridium (Ir)‐dopant, CBP, and Al (Figure 10b).^[^ ^160^ ^]^ PEDOT was the hole injection layer, TAPC was the hole transport layer, CBP was the fluorescent host, Ir‐dopant was the guest with green emission phosphorescence, and the other CBP film where electron transport occurred. ECD was able to modulate the light emission by varying *V*_*G*_ from 4 to 7 V (Figure 10b). In addition, Liu et al. doped the light‐emitting layer with distinct red, green, and blue phosphorescent materials.^[^ ^160^ ^]^ They changed the shape of *G* according to the first letter of the emissive section color, which caused the letters to be displayed following their colors (Figure 10c).^[^ ^160^ ^]^ Other examples of light‐emitting EG‐VOFET‐based prototype displays are shown in Figure 10d,e, in which the *G* electrodes have been patterned to exhibit different symbols.^[^ ^159^, ^161^ ^]^ Figure 10d shows an array composed of *G* electrodes that can be independently turned on, which causes certain regions of the material to emit light to display, for instance, the letters A, T, O, and M.^[^ ^159^ ^]^ Figure 10e shows a device composed of seven *G* terminals that displayed different numbers when switched on at different times.^[^ ^161^ ^]^

The ever‐growing appeal exhibited by IGVTs for cutting‐edge neuromorphic applications makes the light‐emitting EG‐VOFET realization nothing but game‐changing. One step forward, the integration of such a highly efficient, ultra‐compact light‐emitting technology with IGVT multisensory artificial synapses is auspiciously bright. In the same way that IGVTs are already capable of transducing electrical, chemical, mechanical, and optical impulses to EPSC with short‐term and long‐term memories, in the future we envision the integration of light‐emitting IGVTs into artificial synaptic systems to generate excitatory postsynaptic light. This would pave the way for the creation of smart devices based on artificial light‐emitting synapses for applications ranging from smart packaging and tissues, capable of changing their color according to the condition of the product and to the environment or physical and mental states of the user, to dynamic painting of objects and entire places, and intelligent camouflage technologies.

## 4 Neuromorphic Multifunctional Applications

After one and a half decades of research, IGVTs have surpassed other ion‐gating device technologies by achieving faster data processing speeds and more promising memory capabilities. As discussed in Section 3, the IGVTs' low energy consumption, multifunctional integration ability, and multi‐parametric sensing and recording features are critical aspects that can drive and enhance technological advancements. The IGVT research has opened an interesting vista toward manifold applications in artificial synaptic systems, which can already mimic complex functions of the human brain, such as vision, audition, gustation, temperature sensation, and pain perception, among others. Thereby, Section 4 summarizes the most recent signs of progress of the synaptic‐IGVT technology, putting into perspective the implications of neuromorphic research in our society's well‐being. The contents span from the IGVT artificial synapse arrays to the flexible synaptic systems, low‐voltage multisensory systems, and multi‐modal emergent technologies based on synaptic IGVTs. Accordingly, Section 4 is subcategorized into (4.1) pioneering synaptic IGVTs, (4.2) IGVT neuromorphic arrays, (4.3) flexible synaptic IGVTs, and (4.4) synaptic‐IGVT multisensory applications.

### 4.1 Pioneering Synaptic IGVTs

**Figure 11:**
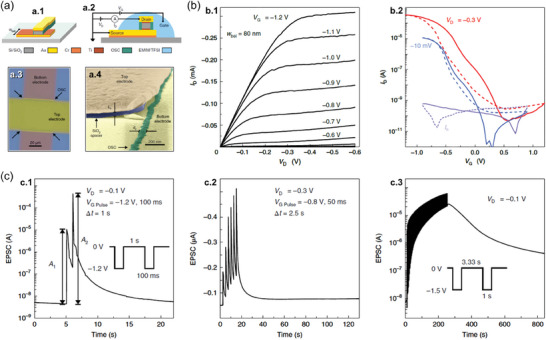
Artificial synaptic behavior in nanoscopic EG‐VOFETs. a) Device structure and microscopy images. a.1) Patterned device base structure (viz. bottom‐ and top electrodes, SiO_2_ spacer, and OSC). a.2) Schematic circuit for the electrical characterization. The materials are labeled at the bottom, whereas *W*_bel_ is the bottom‐electrode width. a.3) Polarization microscopy image of the EG‐VOFET without electrolyte *G*. a.4) False‐color SEM image of the EG‐VOFET. b) EG‐VOFET electrical characteristics: b.1) output‐ and b.2) transfer curves. c) STP and LTP of the EG‐VOFET. c.1) EPSC activated by two −1.2 V‐presynaptic spikes lasting 100 ms each, spaced by one second. c.2) EPSC dynamic response to six *G* spikes of −0.8 V for 50 ms, spaced by 2.5 s apart. c.3) EPSC activated by 73 spikes of −1.5 V lasting one second and spaced 3.33 s for a device featuring only OSC between *S* and *D*. Adapted with permission.^[^ ^69^ ^]^ Copyright 2019, Lenz et al. Published by Springer Nature .

Until 2019, OSCs struggled to perform at their best in highly integrated sub‐100 nm transistors. However, a breakthrough by Lenz et al. that year revealed that electrolyte gating can enhance the electrical performance of organic materials in transistor applications.^[^ ^69^ ^]^ As shown in **Figure** **11**a, they created EG‐VOFETs that featured an OSC length of only 40 nm and a footprint down to the range of 0.01 µm^2^_._ Figure 11a.1 exhibits the schematic illustration of the EG‐VOFET base structure, which was formed by patterned bottom‐ and top electrodes vertically separated by SiO_2_ thin‐film and the lateral OSC that was prepared by spin coating and chemically patterned by reactive plasma. The SiO_2_ layer thickness defined the OSC length. Figure 11a.2 shows the schematic circuit for the electrical characterization, which is completed by adding the electrolyte solution and the external *G* terminal. The device morphology is presented in Figure 11a.3, and in the SEM image of the transistor channel region (Figure 11a.4). The authors stated that the vertical gap was filled with OSC material for all EG‐VOFETs analyzed with SEM.^[^ ^69^ ^]^ Figure 11b exhibits the (b.1) output‐ and (b.2) transfer characteristics of the EG‐VOFETs. The maximum current density for these nanoscopic transistors was in the range of ≈1 MA cm^−2^, whereas the *I*_ON/OFF_ reached up to ≈10^8^. The electrical characterization is completed by testing how long the transistors can maintain ≈1 MA cm^−2^ since Joule heating can substantially degrade the VOFETs. Their long‐term stability experiments revealed that nanoscopic EG‐VOFETs could continuously support ≈1 MA cm^−2^ for 50 min at the least. The positive attributes observed can be ascribed to favorable device geometry, as stated by Lenz et al.^[^ ^69^ ^]^ This is mainly due to the small *W* and *L*, enabling intimate contact between the semiconductor, the *S* and *D* contacts, the SiO_2_ spacer, and the ionic liquid. These components work together as an efficient heat‐dissipation sink.

The nanoscopic EG‐VOFET is a great fit for ANN applications due to its low‐voltage operation, small size, high switching speed, long‐term stability, and use of electrolyte gating.^[^ ^69^ ^]^ To demonstrate its versatility in this field, the pioneering device was used in STP and LTP experiments. Figure 11c.1 shows the EPSC data, in which the second postsynaptic response amplitude was 58 times higher compared to the first one (viz. *A*_2_  =  608 µA versus *A*_1_  =  10.5 µA, respectively). This is due to the remaining‐ion contribution to the succeeding presynaptic pulse (i.e., ions were not randomly redistributed after the initial pulse. To assess LTP formation, Figure 11c.2 exhibits the measurement of a non‐volatile EPSC resulting from multiple −0.8 V pulses lasting 50 ms each, spaced by a 2.5 s inter‐spike interval. Undoubtedly the non‐volatile current represents memory formation. According to Lenz et al., the LTP response can easily be increased in the nanoscopic EG‐VOFETs by decreasing the SiO_2_ width and (consequently) filling out the space with the OSC (Figure 11a.2).^[^ ^69^ ^]^ An EPSC maximum situation is exhibited in Figure 11c.3, in which only the OSC is placed between *S* and *D*. In these devices the ESPC raised by ≈3000 after the last spike and by ≈50 after 10 min. These findings demonstrated the potential of nanoscopic EG‐VOFETs to be utilized as versatile memristive elements featuring tunable STP‐ and LTP relative susceptibility. Furthermore, although Figure 11 illustrates the capability of organic components in nanosized devices to bear 1 MA cm^−2^, Lenz et al. also make it clear that new studies on the electrolytic *G* structuring are necessary, along with investigations on parasitic capacitances involving the electrolyte and *S–D* terminals.^[^ ^69^ ^]^ Recent findings on materials research allow us to point out that the integration of coplanar *G* electrodes, as well as the use of freestanding nanomembranes in IGVT fabrication, could be effective solutions for addressing some of the postulated challenges in this field.^[^ ^19^ ^]^

**Figure 12:**
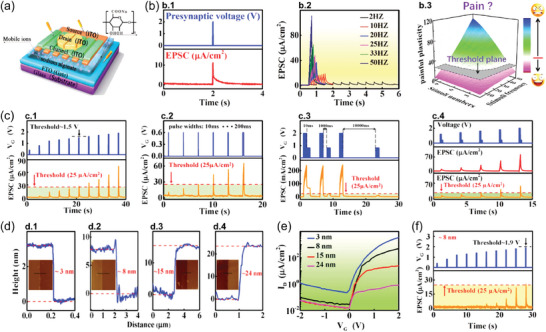
Sub‐10 nm organic‐inorganic EG‐VFET for nociceptor emulation. a) Schematics of the EG‐VFET structure and functional components. b) EPSC and pain threshold: b.1) EPSC for a presynaptic pulse of 2 V lasting 10 ms (*V_D_* = 2 V), b.2) EPSCs for different frequencies (2–50 Hz), b.3) spike‐rate dependent plasticity as a function of stimuli number and frequency (threshold plane: 25 µA cm^−2^). c) EG‐VFET response to electrical pulses: c.1) 10 ms pulse width and 0.4–1.9 V amplitude, c.2) 0.6 V amplitude pulses lasting from 10 to 200 ms, c.3) 2 V amplitude followed by 0.8 V amplitude pulse train with 10 ms‐10 s time interval between the two continuous stimulus trains, and c.4) 1.2–2.0 V amplitude before a set of 0.4 V amplitude pulses. Spikes were spaced by 1 ms. d) Height profiles and AFM images (insets) of the ITO channel edges of d.1) 3 nm, d.2) 8 nm, d.3) 15 nm, and d.4) 24 nm thick samples. e) EG‐VFET transfer curves at *V_D_* = 2 V for different channel thicknesses. f) Pulsed evaluation of 8 nm thick channel EG‐VFET. Adapted with permission.^[^ ^36^ ^]^ Copyright 2019, WILEY‐VCH.

Feng et al. developed an ITO EG‐VFET composed of a ≈3 nm channel.^[^ ^36^ ^]^ The ultrathin device operates in a sodium alginate (SA) biopolymer electrolyte. The organic/inorganic EG‐VFET is capable of mimicking significant aspects of pain perception nociceptors, for instance, aching threshold, memory of preceding traumas, and sensitization and desensitization to ache. **Figure** **12**a exhibits a schematic structure of the EG‐VFET. This vertical transistor has the following three unique features: 1) *L* is simple to tune since it matches the material thickness; 2) the comb‐like *S* and ultrashort channel improve *G* modulation; 3) with the circular top *D*, a uniform current can be achieved because charge carriers move in the channel material at 90 degrees to the substrate.^[^ ^36^ ^]^ The resulting EG‐VFET also displayed high light transmittance (viz. ≈75% in the visible light range). Figure 12b provides the foundation mechanisms that rule the pain‐perception emulation. Figure 12b.1 shows a typical EPSC triggered by a presynaptic spike. The EPSC reaches a ≈12 µA cm^−2^ amplitude and then returns to 0.2 µA cm^−2^. Figure 12b.2 shows the EPSC variations upon 10 stimulus spikes with different spiking frequencies. At 2 Hz the EPSC peak value is low (≈6 µA cm^−2^) even after 10 electrical pulses. However, the increase in frequency enhanced the EPSC amplitude, which presented the highest value at 50 Hz. These findings show that the EG‐VFETs can modulate EPSC using the spike rate, which is a critical property of synaptic plasticity. Such a descending modulatory control of pain‐perception nociceptors is a primary character in the experience of pain in complex biological systems.^[^ ^163^ ^]^ The pain‐perception nociceptor is activated if the harmful stimulus response surpasses a threshold value.^[^ ^164^ ^]^ Feng et al. used this inspiration to define an EPSC threshold, ≈25 µA cm^−2^, in their EG‐VFETs and then mimic pain‐perception nociceptor behavior,^[^ ^36^ ^]^ as shown in Figure 12b.3. The artificial nociceptor may exhibit typical nonpainful behavior characteristics upon stimulus number or frequency yielding EPSC lower than the threshold plane, whereas the exceeding‐threshold stimuli would produce substantial pain.

Ache indicator transference between neurons can be tuned by modifying the pain‐perception nociceptors with specific molecules, channel materials, and receptors.^[^ ^165^ ^]^ Figure 12c shows that the EG‐VFET can mimic the pain‐perception nociceptor behavior.^[^ ^36^ ^]^ For 10 continuous electrical pulses applied to the EG‐VFET *G* (Figure 12c.1), the EPSC did not exceed the threshold level until the pulse amplitude increased to 1.5 V, i.e., similar to the actions of biological nociceptors that increase the response intensity when the harmful stimuli increase.^[^ ^166^ ^]^ A steady step‐by‐step increase was observed in the EPSC upon increasing pulse widths (Figure 12c.2), emulating the biological situation of mild superficial trauma. In such a case, a quick harmful stimulus made the ache imperceptible to the neural system. However, the system interpreted a prolonged harmful stimulus as a hurting sensation.^[^ ^167^ ^]^ The recovery after an injury and the injury severity are two significant aspects when dealing with sensitization,^[^ ^165^ ^]^ and they can be mimicked by the EG‐VFET as well. In Figure 12c.3, EPSCs were acquired by applying a pair of sets of pulse trains, having the first train 50 continuous electric pulses. As noted, this stimulus resulted in a large EPSC (viz. ≈240 µA cm^−2^) that was interpreted as an artificial response to the repeated or particularly intense noxious stimulus.^[^ ^36^ ^]^ Figure 12c.4 shows the effect of the severity of injury on pain sensitization imitated by the EG‐VFET. This sensitization behavior was replicated by applying a set of pulses with amplitudes of 1.2 and 2 V before using subthreshold pulses of 0.4 V amplitude.^[^ ^36^ ^]^ The response signal is shown by the red curve in Figure 12c.4 middle panel. The bottom panel in Figure 12c.4 shows the effect of the initial pulse set on ache sensitization activated by the subthreshold pulse. They found that 0.4 V stimuli cause an EPSC gradual stepwise increase as the first injury stimulus increases.^[^ ^36^ ^]^ This infers a decrease in the threshold value due to a reduced threshold stimulus being needed to cause severe trauma. Such behavior agrees with allodynia and hyperalgesia episodes in nociceptors.^[^ ^165^ ^]^

Feng et al. ultimately succeeded with the implementation of sensitization‐regulated nociceptors using their EG‐VFETs.^[^ ^36^ ^]^ They prepared devices with different *L* values (3, 8, 15, and 24 nm) just by adjusting the thickness of the channel material over its deposition. Figure 12d.1–4 exhibits the corresponding atomic force microscopy (AFM) evaluation of the as‐fabricated ITO films. Figure 12e shows the corresponding transfer curves acquired for the four EG‐VFETs. The differences arising from *L* changes impacted device EPSC, resulting in the apparently continuous increase of the pain‐triggered threshold voltage (*V*_PT_). As pointed out in their article, *V*_PT_  =  1.5, 1.9, 2.2, 2.6 V when the *L* value is 3, 8, 15, and 24 nm, respectively.^[^ ^36^ ^]^ It came out that the EG‐VFETs may have strong responses modulated by sensitization, especially for devices with *L* < 9 nm.^[^ ^36^ ^]^ The artificial sensitization‐regulated nociceptor behavior resembles the biological procedure to adjust the excitable membrane through ion concentration in the cerebrospinal nervous system. Furthermore, it holds great promise in helping ache‐sensitive people minimize their painful feelings by regulating neuronal action.^[^ ^168^ ^]^ In this scenario, the realization of sensitization‐regulated nociceptors based on EG‐VFET can improve bionic medical device efficiency by providing an on‐demand sensitivity to external stimuli.

### 4.2 IGVT Neuromorphic Arrays

**Figure 13:**
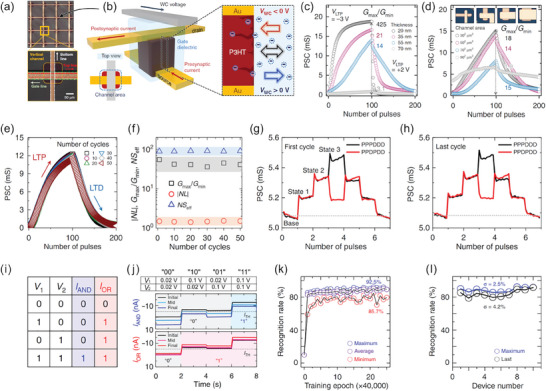
VOECT artificial synapse expandable to a crossbar array. a) Optical microscopy image of the ANN. The inset shows the top view of a device. b) Sketch of the three‐terminal synaptic device at each crossing point of the presynaptic and postsynaptic electrodes in the ANN. The inset illustrates the negative ions of the ion‐gel penetrating or moving out from the free volume of the channel when a negative or positive *V*_WC_ is applied, respectively. c) LTPot/Dep curves for vertical synaptic devices with different P3HT thicknesses under 100/100 potentiation/depression *V*_WC_ pulses of −3 and +2 V. d) LTPot/Dep responses for synaptic devices with various channel areas. The inset shows the change in the channel area. e) 50‐cycle‐LTPot/Dep curve to verify the operational stability of the device. f) *G*_max_/*G*_min_, |*NL*|, and *NS*_eff_ estimated during 50 LTPot/Dep cycles. Comparison of the initial and final PSC cycles. Data were acquired by applying a series of g) regular (PPPDDD) and h) random (PPDPDD) potentiation and depression *V*_WC_ pulses. i) AND and OR logic gates truth table. j) Synaptic array response for training and classification of the logic gates. k) Relationship between the maximum, average, and minimum recognition rates and the number of epochs for ten VOECTs. Panel l) displays the highest and final recognition rates for ten synaptic devices. Adapted with permission.^[^ ^79^ ^]^ Copyright 2020, Choi et al. Published by Springer Nature.

Choi et al. conducted a pioneering study utilizing VOECTs for high‐performance and high‐density neural network development.^[^ ^79^ ^]^ They proposed a crossbar array structure for an ANN that was composed of vertical three‐terminal units with excellent synaptic characteristics (**Figure** **13**a). Figure 13b shows the device unit composed of a 100‐nm‐thick P3HT channel between the pre‐ and postsynaptic contacts. The weight‐control (WC) material was deposited covering the whole structure and was made of ionic liquid plus poly(vinylidene fluoride‐co‐hexafluoropropylene) (PVdF‐HFP).^[^ ^79^ ^]^ The synaptic characteristics were acquired by investigating the channel conductance since changes in its value are similar to the operation of a biological synapse.^[^ ^169^, ^170^, ^171^ ^]^ The channel conductance can be controlled by applying a negative or positive WC voltage (*V*_WC_), which makes ions move in or out, respectively, from the free volume of the P3HT layer, as shown in the inset of Figure 13b. A typical transfer curve for the vertical device was acquired by applying a fixed *V_D_* (*V*_pre_), a *V*_WC_ pulse, and by monitoring the PSC between *S* and *D* terminals. Also, the devices showed EPSC and IPSC responses as PSC increased under the application of negative *V*_WC_ or decreased under positive *V*_WC_. The PSC value was maintained for 50 s and did not recover the beginning value due to the remaining ions in the P3HT material. Furthermore, the relaxation times of rapid and slow phases estimated for the vertical synaptic device were compatible with biological synapses, 87 and 1762 ms, respectively. The authors were also successful in estimating the nonlinearity (*NL*), dynamic range (ratio between the maximum conductance value and the minimum one, *G*_max_/*G*_min_), and number of states (*NS*_eff_) from LTPot/Dep curves.^[^ ^79^ ^]^ Such properties are important for the learning and recognition processes necessary for an ANN. The device architecture was optimized to deliver the best values for *G*_max_/*G*_min_, *NL*, and *NS*_eff_, by tuning both channel thickness and area. Figure 13c shows the LTPot/Dep curves for devices composed of P3HT channels of 50 × 50 µm^2^ area and thicknesses from 20 to 95 nm. For the LTPot/Dep measurement, 100 potentiation *V*_WC_ pulses of −3 V followed by 100 depression *V*_WC_ pulses of +2 V were applied with a 2 Hz frequency and 50 ms width. Due to the vertical channel, the highest maximum conductance (18.9 mS) was found for the thinnest channel, and the devices with P3HT thicknesses of 20, 35, and 55 nm exhibited *G*_max_/*G*_min_ > 10, the minimum value needed for pattern recognition. However, the 55‐nm‐thick P3HT channel exhibited the best responses for both regions in LTPot/Dep curves leading to the lowest |*NL*| value (0.6/2.3) and the highest *NS*_eff_ (96/72). Furthermore, the channel area influence on LTPot/Dep responses was evaluated by changing the metal line width and maintaining the channel thickness at 55 nm (Figure 13d).^[^ ^79^ ^]^ The device with a 30 × 30 µm^2^ channel exhibited the highest *G*_max_/*G*_min_ value. However, the VOECTs with channel areas of 50 × 50 µm^2^ displayed low |*NL*| values and high *NS*_eff_, in addition to a suitable *G*_max_/*G*_min_.^[^ ^79^ ^]^ Therefore, Choi et al. chose devices with a channel of 55 nm thickness and an area of 50 × 50 µm^2^ to investigate their operational stability envisioning ANN application. Figure 13e shows the stability of 50 PSC cycles acquired by applying 10 000 potentiation and depression pulses. Figure 13f shows that *G*_max_/*G*_min_, |*NL*|, and *NS*_eff_ properties varied by 1.1%, 1.4%, and 6.4%, respectively, for the 50 cycles. Regular (PPPDDD) and random (PPDPDD) conditions of potentiation and depression *V*_WC_ pulses were applied to examine the reliability of the PSC response. Figure 13g,h shows that the first and last PSC cycles varied <1% from baseline for each pulse cycle.^[^ ^79^ ^]^

Choi et al. trained binary AND and OR onto a simple synapse array to mimic a neural network composed of three input neurons (*V*_1_, *V*_2_, and a bias voltage *V*_b_), two output neurons (currents for the binary logic operations, *I*_AND_ and *I*_OR_), and six synapses connecting them (represented by conductance values).^[^ ^79^ ^]^ For the learning process, applied input voltages led to an output current vector that was the inner product between the conductance matrix and the input voltage vector. Afterward, the output currents were compared with a threshold value (*I*_TH_) and classified as “0”, for output currents less than *I*_TH_, and as “1”, for output currents higher than *I*_TH_. Then the conductance values were updated by a *V*_WC_ to minimize the difference between the output value and those shown in the truth table of Figure 13i. Figure 13j exhibits the output currents before the training process close to the *I*_TH_, and then shows the output currents for inputs “00”, “10”, “01”, and “11”. The differences between the output currents and the *I*_TH_ of the AND and OR operations after the learning process provided AND and OR logic gate functions for the synapse array.^[^ ^79^ ^]^ The implementation of a simple synapse array into a complex ANN has also been successfully demonstrated, which may pave the way to developing dense neural networks with improved performance. The size of the neural network was 400 × 200 × 10, and it was composed of ten devices.^[^ ^172^ ^]^ For training and recognition, the authors employed the Modified National Institute of Standards and Technology (MNIST) dataset. Figure 13k shows the recognition rate for the ten devices every 1 epoch consisting of 40 000 training processes. The maximum recognition rate was 92.5%, while the minimum was 85.7%. In addition, Figure 13l exhibits the recognition rate variation for device‐to‐device. The maximum recognition rates had a standard deviation of 2.5%, which changed to 4.2% after the last training process.^[^ ^79^ ^]^

**Figure 14:**
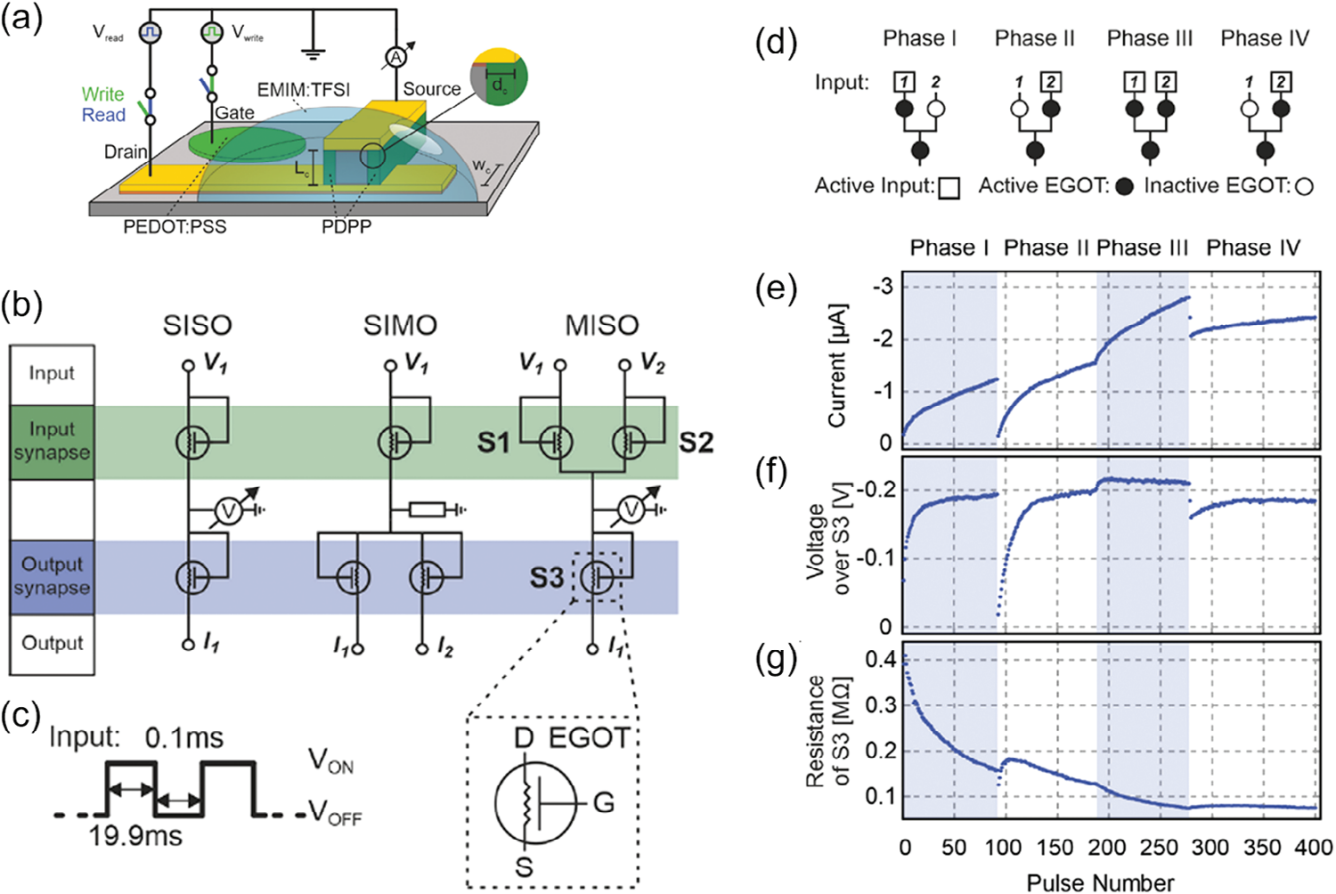
Brain‐inspired, low‐voltage, interconnected EG‐VOFET array. a) Device geometry and measurement circuit. b) Circuit configuration with the three synapses S1–S3. c) Input signal parameters: turn‐off voltage = 0 V, and turn‐on voltage < −0.6 V. d) Activation of the inputs during phases I–IV. e) Output current. f) Voltage drops across the EG‐VOFET. g) EG‐VOFET resistance. Adapted with permission.^[^ ^81^ ^]^ Copyright 2022, American Chemical Society.

In a similar work, Eckel et al. mimicked biological synaptic functions via an interconnected system of EG‐VOFETs (**Figure** **14**a).^[^ ^81^ ^]^ Namely, the authors explored information transfer over multiple EG‐VOFETs (similar to the human brain) where each device was supposed to mimic one synapse. To this end, the *D* and *G* contacts of each device were connected to obtain the PPF effect. This setup was aimed at each pulse prompting a change in the channel conductivity (caused by the penetration of ions) leading to a current response at the *S*. In this pursuit, three circuit configurations were prepared (Figure 14b), aimed at realizing information accumulation from autonomous inputs and plasticity effects at the output end reminiscent of neuron cells. This led to a history‐dependent information transfer over multiple synapses (Figure 14c–g).

The measurements were performed in four phases to demonstrate the ability of EG‐VOFETs to mimic three synapses (S1 – S3), as shown in Figure 14d. In the first two phases (phase I and phase II), input signals were applied exclusively to S1 and S2, respectively. Because of the high resistance of S2, the current magnitude dropped sharply (Figure 14e), which also caused a decrease in voltage drop over S3 (Figure 14f). On the other hand, since ions from pulses in phase I were still present, the devices' memory effect (Figure 14g) caused the S3 resistance to remain comparatively constant. In phase III, the summation effect of inputs (similar to biological neurons) resulted in further increasing the current conductance. A memory effect for S3 resistance appears in the passage from phase III to IV, while the current/voltage drops decreased to lower values, prompted by switching off one input path. In summary, these measurements demonstrate the interconnectivity of EG‐VOFETs (also a basic requirement of complex circuits) and how this can be used to mimic fundamental synaptic functions.

Neuromorphic processors are composed of artificial synaptic devices that enable fast, parallel computing. Most current research on artificial synaptic units has focused on p‐type polymers to result in organic synapses, with restricted studies done on n‐type materials judged for their sub‐estimated performance. Nonetheless, the development of n‐ and p‐type materials is necessary to improve the IGVT versatility, as well as to enable the creation of more efficient complementary neural network circuits. To address this issue, different device structures and molecular modification strategies have been employed to improve the performance of n‐type materials to mimic artificial synapses.^[^ ^173^, ^174^ ^]^ Xie et al. have developed an all‐solid‐state IGVT composed of an organic dielectric based on Li^+^ and with a channel made of an n‐type donor‐acceptor conjugated polymer. The device exhibited nonvolatile conductance modulation at a low voltage, reached high current density, and mimicked the LTP and PPF synaptic functions, all with an energy consumption comparable with devices based on p‐type materials.^[^ ^173^ ^]^ In addition, Wang et al.^[^ ^174^ ^]^ have demonstrated that mixing complementary molecular building blocks is a useful strategy for designing materials with efficient transport of mixed charges, capable of being used in cutting‐edge applications when incorporated into sophisticated device architectures. For this purpose, they reported an n‐type fluorinated lactone‐based polymer that exhibited suitable properties as an OECT channel material, such as a high mobility‐by‐volumetric‐capacitance product (*µC* ^*^, where *µ* is the OSC field‐effect charge‐carrier mobility and *C* ^*^ is the volumetric capacitance), low threshold voltage, and fast switching speed. They employed such a polymer in a VOECT architecture composed of an Au *G* and an [EMIM^+^][TFSI^−^] electrolyte, capable of mimicking volatile and nonvolatile synapses exhibiting STP and LTP features, respectively.^[^ ^174^ ^]^

### 4.3 Flexible Synaptic IGVTs

**Figure 15:**
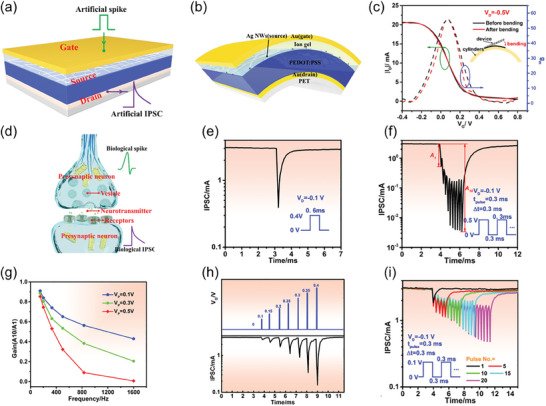
Synaptic VOECTs, flexible VOECTs, and their artificial synapse applications. a) Illustration of the vertical device architecture, and input (viz. artificial spike) and output (viz. artificial IPSC) electrical signals. b) Illustration of the PET‐based flexible VOECT. c) Transfer and transconductance curves for the PET‐based VOECT prior to and after a 10 mm radial bending test. The inset exhibits the schematic of the bending tests. d) Illustration of the chemical synapse working principle: biological spike and biological IPSC, as mimicked by the VOECT. e) IPSC response to a pair of pulses 0.6 ms spaced. *A* *_x_* represents the peak IPSC for the pulse number *x*. f) Dynamic response of IPSC for a set of 10‐presynaptic‐spike set. Each pulse was spaced by a 0.3 ms interval time. g) PSC gain for a set of presynaptic pulses. The spikes had distinct frequencies. h) IPSC response for distinct amplitude presynaptic pulses lasting 0.5 ms each. i) IPSC response to sets of presynaptic spikes composed of distinct numbers of pulses. Adapted with permission.^[^ ^40^ ^]^ Copyright 2020, American Chemical Society.

OECTs have garnered significant attention for their diverse applications in flexible, stretchable, and wearable electronics‐ fostering the development of biosensors, digital logic circuits, and artificial synapses. Unfortunately, most OECTs still employ planar *S*‐channel‐*D* configurations that may be detrimental to many flexible applications. Furthermore, planar OECTs currently available employ relatively long OSC polymers that feature *L* values with up to several tens of micrometers. Both factors restrict the flexible device performance and result in lower OECT densities for flexible active‐matrix applications. The IGVT architecture may tackle such issues. Yan et al. reported on the design, fabrication, and characterization of flexible VOECTs that featured a nanoscale *L* of ≈100 nm.^[^ ^40^ ^]^ **Figure** **15**a exhibits the schematics of the proposed device architecture. The devices displayed impressive qualities, such as an *I*_ON_ > 20 mA at a mere ≈0.5 V range, a quick dynamic response (< 300 µs), and a remarkable transconductance value (68.88 mS).^[^ ^40^ ^]^ The remarkable electrical performance is credited to the vertical device design featuring *L* determined by the thickness of the semiconductor material. The tolerance of the OECTs to mechanical perturbations was also investigated by employing flexible substrates. Figure 15b illustrates the VOECT fabricated on a flexible polyethylene terephthalate (PET) base. Figure 15c shows transfer and transconductance curves for VOECTs prior to and after of bending processes. The mechanical tests unaffected the electrical results, which were comparable to those acquired for their inflexible counterparts. For the bending essays, VOECTs were fastened to 20 mm‐diameter cylinders, as illustrated in the inset of Figure 15c.^[^ ^40^ ^]^

VOECTs are promising devices as neuromorphic single units and in ANNs due to their low working voltage, quick responses, and reduced sizes. Figure 15d depicts the chemical synapse working principle with its signals that can be mimicked by the PEDOT:PSS‐based VOECTs (as in the schematics of Figure 15a). One may notice that the *G* mimics the presynaptic neuron, whereas the PEDOT:PSS channel and the *S*‐ and *D* electrodes mimic the postsynaptic neuron. Figure 15e shows the representative IPSC behavior. According to Yan et al., Li^+^ can penetrate the PEDOT:PSS layer under a positive and pulsed presynaptic *V_G_* application.^[^ ^40^ ^]^ The IPSC sudden decay was explained by the compensation of the sulfonate groups by the penetrated cations. This effect caused the conductive PEDOT^+^ to reduce to its insulating form. After the removal of the *V_G_*, the IPSC slowly recovered its beginning value because the cations returned to the bulk electrolyte. Such an operation resembles IPSC responses at a real inhibitory synapse.^[^ ^40^ ^]^

To mimic the temporal processes that take place in the synaptic cleft, presynaptic voltage spikes can be applied as input stimuli in the VOECT's *G*. Yan et al. investigated temporal correlation in their devices by applying sets of 10 pulses.^[^ ^40^ ^]^ They employed a pulse duration of 0.3 ms with a signal amplitude of 0.5 V, whereas the input frequency ranged from 200 to 1600 Hz.^[^ ^40^ ^]^ Figure 15f shows that the application of the pulse sets caused moderate modifications in the depressive conductance in the IPSC. In addition, IPSC gain was also estimated by dividing the peak value of the last IPSC peak in the set by the peak value of the first peak in the set (*A*_10_/*A*_1_). The frequency increments up to 1600 Hz reduced the IPSC gain (Figure 15g).^[^ ^40^ ^]^ The investigation of the inhibitory behavior was performed by applying *V_G_* pulses with 0.1 to 0.4 V amplitudes (Figure 15h). The IPSCs displayed maximum amplitude at the pulse ending. The amplitude increments with the presynaptic signal rising were comparable with the inhibitory processes in biological synapses.^[^ ^40^ ^]^ Considering an increasing pulse number, Figure 15i shows that IPSC had inferior amplitude, requiring additional time to restore the early current level. Such behavior indicates a change from short‐ to long‐term memory.^[^ ^155^ ^]^

**Figure 16:**
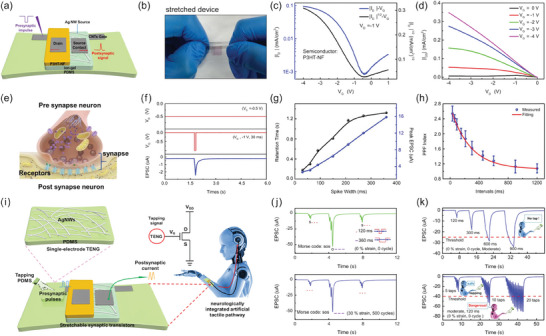
Stretchable IGVTs for neuromorphic applications. a) Sketch of IGVT fabricated on a PDMS substrate used for the synaptic essays. A similar architecture, but with a cross‐linked PVA layer instead of ion‐gel, was used for stretching processes. b) Photograph of a real device under stretch test. c) Transfer and d) output curves for the vertical transistor with a PVA layer and on a non‐stretched state. e) Illustration of a chemical synapse. f) EPSC resulted from a single spike signal. g) Retention time and peak EPSC for different spike widths. h) Pulse interval influence on PPF index for unstretched devices. i) Scheme and the circuit diagram for a self‐powered integrated artificial tactile pathway. j) EPSC signals are triggered by a single tactile stimulus and can be related to the international Morse code for "SOS". k) The upper panel shows the EPSC signal obtained with a single touch spike at different times. The lower panel shows EPSC signals acquired with different numbers of touch spikes with moderate strength for 120 ms. Adapted with permission.^[^ ^175^ ^]^ Copyright 2021, Elsevier.

Wang et al. have fabricated and investigated the vertical stretchable synaptic transistors for flexible neuromorphic systems.^[^ ^175^ ^]^ **Figure** **16**a illustrates the organic transistors fabricated on a polydimethylsiloxane (PDMS) substrate. The devices comprised carbon‐nanotube (CNT) *G* and *D*, AgNW *S* network, and a P3HT nanofibrils (NF) semiconducting layer. The dielectric layer was composed of a cross‐linked polyvinyl alcohol (PVA) insulating layer for the stretching essays and replaced by an ionic‐gel electrolyte material containing polyacrylonitrile (PAN) polymer plus bis (trifluoromethane) sulfonamide lithium ([Li^+^TFSI^−^]) for the neuromorphic operation.^[^ ^175^ ^]^ Figure 16b shows a real device subjected to a stretching essay. The transfer and output characteristics for the unstretched transistors are shown in Figure 16c,d, respectively. Wang et al. observed typical p‐type transistor curves with a saturated region and with a low operating voltage of −0.7 V.^[^ ^175^ ^]^ In addition, the electrical performance of the devices was stable after submitting and releasing them to stretching strains. It is noteworthy that for real applications, stretchable devices need to maintain their functionalities even when they are subjected to mechanical deformation. Accordingly, the effect of different stretched strains from 0 to 20% on the device performance was investigated by analyzing the *I*_ON_‐ and *I*_OFF_ densities, and the normalized *SS*. Changing the stretching strain from 0 to 20% increased by ≈43.8% the ON‐state current density and decreased *SS* by ≈16.1%. These findings show greater ease in the vertical charge transport in the devices, which was due to the improvement of the π–π stacking “face‐on” alignment in the P3HT‐NF film made possible by the elongation stress.^[^ ^175^ ^]^

In the biological system depicted in Figure 16e, presynaptic neurons transmit signals to postsynaptic ones by chemical synapses. Thus, neurotransmitters are released across the synaptic cleft and induce postsynaptic potential in the postsynaptic neurons. Three‐terminal devices can mimic biological synapses when postsynaptic signals (*I_D_* with a driving *V_D_*) are obtained when charge carriers and ions accumulate near the dielectric/semiconductor interface by applying different presynaptic *G* pulses. Figure 16f shows a single −1.0 V, 30 ms *G* pulse triggering an EPSC spike for the unstretched device, with behavior resembling the short‐term memory EPSC response for biological synapses. Figure 16g shows the influence of the spike width of the *G* pulses on the retention time and EPSC amplitude, which were higher for larger presynaptic pulse widths.

The receptivity of biological synapses to process and learn information is indicated by PPF,^[^ ^176^, ^177^, ^178^ ^]^ which is acquired using two pulses spaced within a short period. Figure 16h shows the PPF index for the unstretched device decreasing with spike interval time increasing since the anions accumulated by the first spike have more time to diffuse away. In addition, synaptic characteristics and electrical performance of the IGVTs were stable under stretching strains. Finally, Wang et al. showed a self‐powered neurologically integrated system comprised of a triboelectric nanogenerator (TENG) and an IGVT, which simulated tactile sensing and processing. Figure 16i illustrates the integrated artificial tactile pathway and its circuit diagram. The TENG electrode generated presynaptic pulses that were transferred to the artificial synapse, leading to excitatory postsynaptic signals. In addition, the international Morse code and human skin pressure ulcer were imitated using the artificial tactile pathway to demonstrate its suitability for wireless communication and sensory interaction with skin touch. By pressing and releasing the integrated system, different amplitudes of EPSC were acquired and could be related to the letters of the English alphabet. Figure 16j shows the “SOS” emergency signal expressed by the unstretched system (upper panel) and by the tactile pathway subjected to the stretched strain (bottom panel). Finally, for the sensory skin touch‐interaction essay in Figure 16k, the presynaptic spikes were the tactile signal, and an EPSC current was defined as the threshold for the tactile responses. For a unique pressure and releasing procedure (upper panel), an EPSC current peak above 25 µA indicated that the body was not being touched, whereas a higher current meant that touching still existed and attention should be kept for possible pressure ulcers. For situations with an increase in pressure and release times, the threshold was defined as 40 µA. The peak current of the EPSC was above the threshold when pressing and releasing more than ten times, which indicated that pressure ulcers could be induced, triggering a dangerous signal. Therefore, the findings demonstrate the vast potential of IGVTs in wireless communication, neuromorphic systems, and intelligent robotics.^[^ ^175^ ^]^

### 4.4 Synaptic‐IGVT Multisensory Applications

**Figure 17:**
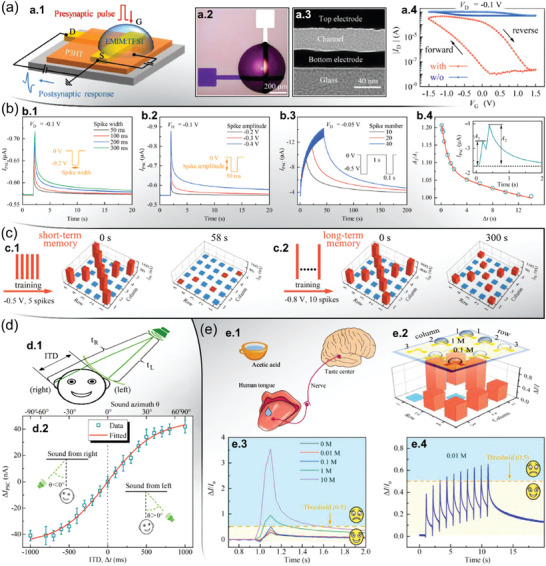
Multisensory artificial synapse based on EG‐VOFET. a) EG‐VOFET architecture and electrical characteristics. a.1) Synaptic transistor architecture with the ionic *G* dielectrics and electrical measurement sketch. a.2) EG‐VOFET optical microscopy image. a.3) TEM image for a cross‐section of the dry device. a.4) Transfer curves for a bare device and a device with the cross‐linker. b) Synaptic functions emulated by the EG‐VOFET. b.1) PSC triggered by *V_G_* = −0.2 V pulses with various spike widths. b.2) PSC triggered by 50 ms‐duration *V_G_* pulses. b.3) Relaxation performance for 10, 20, and 40 spikes (0.1 s duration, 1 s interval). b.4) PPF index (i.e., *A*_2_/*A*_1_) depending on the pulse interval. The inset shows PSC for two 0.2 s‐presynaptic pulses. c) Synapse array for image memorizing. c.1) Short‐term memory states were obtained by applying five training −0.5 V amplitude, 0.1 s duration, 0.1 s interval pulses (viz. "X" shape). c.2) Ten −0.8 V amplitude, 0.5 s duration, and 0.1 s interval spikes led to a long‐term memory state. d) Spatiotemporal processing using EG‐VOFET artificial synapse for sound detection. d.1) Schematic illustration of sound position, noticed by the ears along with the brain. d.2) Postsynaptic difference in response to the time interval and azimuth sound. e) EG‐VOFET artificial tongue and AA detection. e.1) AA detection schematic illustration. e.2) Illustration of the tongue array in the presence of 0.1 and 1 M AA‐concentration (top), which led to the taste map (bottom). e.3) Device response to −0.3 V input in the presence of AA solutions with concentrations ranging from 0 to 10 M. e.4) EG‐VOFET response to a constant stimulus and using 0.01 M AA solution. Adapted with permission.^[^ ^56^ ^]^ Copyright 2022, Wiley‐VCH.

The IGVT's multi‐parametric properties are remarkable tools to control the plethora of functions accessible in ion‐gating devices. Among the IGVT envisioned functionalities to fine‐tune the device in a multisensory and multi‐modal fashion are electrical and ionic switching, mixed‐ionic‐electronic conduction, thermoelectric energy conversion, and photocurrent generation, to mention a few. Liu et al. have provided a pioneering realization based on the EG‐VOFET technology, viz. a multisensory artificial synapse.^[^ ^56^ ^]^ **Figure** **17**a.1 shows the schematic structure of the EG‐VOFET with electrolytic *G*. The top and bottom electrodes (viz. *S* and *D*, respectively) were responsible for the artificial post‐neuron signal, whereas the *G* dielectric acted as an artificial pre‐neuron counterpart. The semiconducting vertical channel is composed of P3HT 1,6‐bis(trichlorosilyl) hexane.^[^ ^56^ ^]^ Figure 17a.2 shows the optical microscopy image of the EG‐VOFET. Figure 17a.3 displays a transmission electron microscopy (TEM) image for a cross‐section of the EG‐VOFET without the electrolyte, evidencing the ≈30 nm thick OSC. Figure 17a.4 shows the transfer characteristics of the EG‐VOFETs displaying pristine (blue curve) and cross‐linked (red curves) P3HT. An evident hysteresis curve, along with an intense *I*_ON/OFF_, was verified for the cross‐linked device, allowing the emulation of synaptic plasticity functions.^[^ ^56^ ^]^ Figure 17b.1–3 exhibits EPSC resulting from the OSC charge transport at the EG‐VOFET's ON state, as induced by the anions migration to the channel interface caused by the *V_G_* application. A clear EPSC increasing trend was observed with increasing spike duration (Figure 17b.1). When a short spike (viz. 50 ms) was applied, EPSC changed temporarily, quickly returning to its initial value, similar to biological synapse STP behavior. The EDL formation dynamics provided the electrostatic coupling that led to the channel conductance volatility.^[^ ^56^ ^]^ In contrast, Figure 17b.2 shows that, by enhancing the pulse amplitude from 0.2 to 0.4 V, EPSC increased and needed more time to regress to its primary value, indicating a possible transition to LTP.^[^ ^56^ ^]^ To mimic the release of plenty of neurotransmitters from the presynaptic membrane to the cleft due to external triggers,^[^ ^179^ ^]^ the EG‐VOFET was generated by spike trains of −0.5 V amplitude, 5 s duration presynaptic signals with 10, 20, and 40 pulses each (Figure 17b.3). When more spikes were applied to the *G*, EPSC improved significantly as a function of the number of pulses. Figure 17b.4 exhibits the PPF characteristics of the EG‐VOFET. Highly amplified EPSCs were generated by a pair of consecutive −1 V amplitude, 0.1 s duration spikes spaced by 0.2 s (Figure 17b.4‐inset). The PPF index, *A*_2_/*A*_1_, decreased gradually with increasing time intervals, indicating that the ions moved by the initial pulse had sufficient time to diffuse to their initial conditions when increasing time intervals.^[^ ^56^ ^]^

The artificial synapse memory plasticity was assessed by an image learning and memorizing experiment using a 5 × 5 EG‐VOFET array that was in a low conductivity condition previous to the application of electrical triggers.^[^ ^56^ ^]^ As shown in Figure 17c, an image with an “X” was employed for memory instruction. The image “X” was then inputted to the synapse array with a pulse train composed of five pulses with −0.5 V amplitude, 0.1 s duration, and 0.1 s interval (Figure 17c.1). The enhanced current rapidly dropped to the initial state after 58 s, indicating a short‐term memory behavior. In Figure 17c.2 the pixels were trained by applying 10 stronger presynaptic stimuli with −0.8 V amplitude, 5 s duration, and 0.1 s interval. The image “X” was readable on the array even later than 300 s of the final instruction input, indicating long‐term memory behavior. The findings showed that the EG‐VOFET generated artificial synapses capable of being employed for low voltage, highly efficient image learning and memorizing.^[^ ^56^ ^]^

Perceiving sound location is crucial for an audio‐sensing nervous system to obtain surrounding data. Thus, a pair of ears is useful as it can localize noises by interaural time difference (ITD).^[^ ^180^ ^]^ Figure 17d.1 illustrates the conversion of the distinct paths of the sound waves to arrive at two ears to ITD. To estimate the ITD, the time difference between the waves reaching the right ear (*t*_R_) and the left ear (*t*_L_) was calculated. An ANN was created to mimic the azimuth‐sensing task displayed by the brain.^[^ ^56^ ^]^ Figure 17d.2 exhibits the EPSC variation as a function of the ITD and sound azimuth. The EPSC values in this plot are concentrated in the first and third quadrants, providing the means to achieve spatiotemporal localization.^[^ ^56^ ^]^ The negatively increasing EPSC, ITD, and azimuth in the third quadrant showed that the sound originated from the right, whereas the increasing ones in the first quadrant indicated the sound originated from the left. The findings show that EG‐VOFETS can emulate spatiotemporal information processing activities of the human brain.^[^ ^56^ ^]^

In addition to sound perception, the synaptic EG‐VOFET was applied as an artificial tongue to sense acidity (Figure 17e.1).^[^ ^56^ ^]^ In this application, the ionic liquid worked like a thin coating of saliva for the tongue, where the response to acidity was tested by adding different concentrations of acetic acid (AA). For the detection, a *V_G_* pulse of −0.3 V amplitude and 100 ms duration was employed. Figure 17e.2 displays a 3 × 3 matrix of artificial gustatory pixels responding to 0.1 and 1.0 M AA on the specific spots and presenting taste mapping. Figure 17e.3 exhibits the artificial tongue response to distinct AA concentrations. AA contents surpassing standard tolerance (0.1 M) led to a painful manifestation.^[^ ^56^ ^]^ As noted, the response is more intense for increasing acidity stimuli, which would result in a longer injury recovery. The effect of continuous AA stimulus on the tongue is depicted in Figure 17e.4. The EPSC signal gradually increased even for low acidity (i.e., 0.01 M), exceeding the response threshold level after 3–4 pulses and demonstrating that EG‐VOFETs can alert pain sensation when the stimulus is sufficiently strong. The artificial tongue based on EG‐VOFETs displayed, therefore, a behavior similar to the human tongue licking different AA amounts.^[^ ^56^ ^]^ In summary, the findings endorse that EG‐VOFETs are reliable candidates for integrating neuromorphic systems by mimicking the sensory recognition of living organisms.

Among the recent advances investigated to develop IGVTs with multisensory memory computing functionality leading to improved sensory perceptual efficiency in electronic devices is, for instance, the study by Liu et al. They introduced a novel vertical tribo‐transistor (VTT) device that combines a TENG and a VOFET to enable multi‐sensing, memory, and computing functions while integrating various sensory inputs.^[^ ^181^ ^]^ The VTT device configuration featured MXenes as a versatile material serving as the top electrode of the TENG, the *S* electrode of the transistor, and the light collection layer for multisensory information. This design enabled the VTT to modulate ionic migration and the Schottky barrier through electrostatic induction and tribo‐potential, amplifying triboelectrification‐based sensory data into *S–D* current in a self‐energy transducing manner. This enhancement results in a 711‐fold increase in sensitivity compared to a single TENG device. By integrating the VTT with a robot hand, the system generated artificial conscious responses, successfully controlling the robot hand's open angle in response to different sensory stimuli, thereby improving event accuracy. Moreover, the researchers developed a multi‐model emotion recognition system capable of detecting and distinguishing typical moods. Indeed, the self‐powered VTT device exhibits significant promise for next‐generation high‐performance in‐sensor‐memory‐computing artificial intelligence systems and human‐computer interaction interfaces.

**Figure 18:**
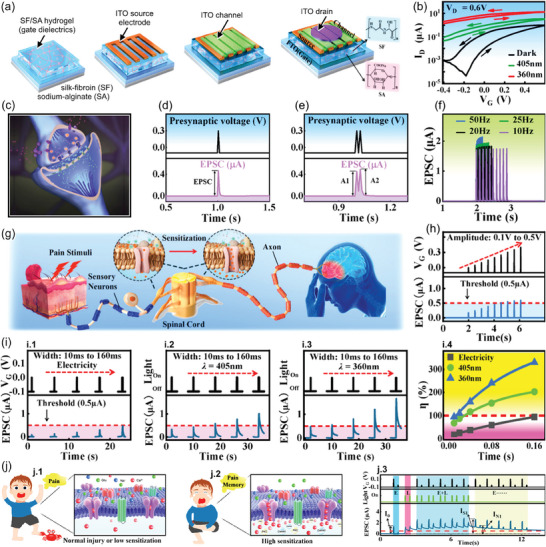
Low‐voltage sub‐10 nm photo‐active EG‐VFET for pain‐sensitization enhancement emulation. a) SF/SA hydrogel‐based vertical transistor manufacturing steps. b) ITO EG‐VFET transfer characteristics acquired under dark, and 405‐ and 360 nm radiation. c) Biological synapse schematics. d) EPSC reaction measured using a 10 ms duration presynaptic spike. e) PPF activated by 10 ms interval presynaptic spikes. f) EPSC responses to 10 spikes with different frequencies. g) Diagram exhibiting pain perception course and the peripheral sensitization mechanism. h) Subsequent electrical spikes imposed on the EG‐VFET with various spike amplitudes. i) Pain perception mechanism at increasing spike durations of i.1) electric pulse, and i.2) 405‐ and i.3) 360 nm laser. The *η* value is plotted as a function of spike duration in panel i.4). j) Illustration of a child pinched by a crab and the central j.1) low‐ and j.2) high sensitization behaviors occurred in his body. Sensitization augmentation treated j.3) from an ordinary wound to a severe condition resulting from Pavlovian instruction. Adapted with permission.^[^ ^38^ ^]^ Copyright 2023, Royal Society of Chemistry.

Paving the way for the research in neuromorphic optoelectronics based on IGVTs, Su et al. designed and characterized a new type of sensory EG‐VFET that exhibited photosensitivity and could emulate pain sensitization through training.^[^ ^38^ ^]^ Their approach was based on the use of an electrolyte based on silk‐fibroin (SF). The fabricated device displayed a 9.6 nm channel and operated at an exceptionally low voltage of ≈0.6 V. It is worth mentioning that the EG‐VFET had several advantages, including the utilization of the protonic SF/SA hydrogel as the *G* dielectric, the ability to easily adjust the ITO film thickness (which determines *L*), and a lower power consumption. Additionally, provided by its high transparency,^[^ ^36^ ^]^ the transistor could perform various photoelectronic sensory functions. **Figure** **18**a illustrates, from the left to the right, the main steps of the device manufacture and the as‐fabricated EG‐VFET structure as well. The EG‐VFET transfer characteristics are shown in Figure 18b. For the dark condition, a clear anticlockwise hysteresis took place arising from the protonic low‐speed relaxation in the SF‐based hydrogel,^[^ ^182^, ^183^ ^]^ whereas *I*_ON/OFF_ was ≈10^4^. The transfer curves obtained under 405 and 360 nm laser illumination (Figure 18b) show a strong photo‐response current along with a noticeable hysteresis window, which is the driving force for achieving sophisticated neuromorphic capabilities.^[^ ^184^ ^]^ Figure 18c illustrates the biological synapse where the action‐potential‐driven interaction between neurotransmitters and unambiguous receptors (at its postsynaptic membrane) causes EPSC or IPSC.^[^ ^72^ ^]^ Given the ion‐gating properties of the photo‐sensible EG‐VFETs, the characteristics of synaptic plasticity can be successfully realized. EPSC can be stimulated by *V_G_* spikes (viz. 0.4 V amplitude), reaching a 0.47 µA peak value (Figure 18d). Additionally, PPF was effectively emulated in the EG‐VFETs (viz. *A*_1_ and *A*_2_ current in Figure 18e) by two identical 0.4 V‐amplitude spikes. In Figure 18f, the EPSC values were displayed for continuous spike trains with varying stimulus frequencies (viz. 10, 20, 25, and 50 Hz). Each spike train consisted of ten consecutive stimuli with a 0.4 V amplitude, 10 ms spike duration, and 10 ms interval. It was evident that as the stimulus frequency increased, the EPSC values also increased and reached their peak at 50 Hz. The human brain's pain‐awareness and peripheral‐sensitization mechanisms are illustrated in Figure 18g. Aching discomfort begins with nociceptor activation which is dissociative axon terminations.^[^ ^185^ ^]^ These receptors can only be activated when a noxious stimulus, such as a strong chemical or mechanical stimulation, surpasses a certain limit (i.e., a specific threshold).^[^ ^186^ ^]^ The EG‐VFET can mimic this pain perception process,^[^ ^38^ ^]^ as shown in Figure 18h. By applying nine continuous electrical spikes within a 0.1–0.5 V amplitude range, the EPSC response exceeded the threshold current for signal amplitudes superior to 0.35 V. The findings of Su et al. evidence that EG‐VFET‐based nociceptors are feasible and fully operational at an ultralow voltage regime of ≈0.6 V.^[^ ^38^ ^]^

Our skin serves as a crucial protective barrier against external factors such as electrical and light stimuli. Excessive exposure to strong UV radiation can lead to photodamaging dermatosis in humans, both directly and indirectly.^[^ ^187^ ^]^ This light‐to‐pain perception and sensitization behavior can also be mimicked by EG‐VFETs, as shown by Su et al. (Figure 18i).^[^ ^38^ ^]^ During their experiments, five electrical‐ and light‐spike sets (10–160 ms duration) were applied to the EG‐VFET (Figure 18i.1–3). The light spikes displayed 255 mW cm^−2^ power intensity. The EPSC responses increased gradually with the electrical spike duration increase but did not exceed the threshold current (Figure 18i.1). It appears that prolonged stimulation may enhance the pain perception abilities of nociceptor neurons.^[^ ^188^ ^]^ In the case of the 405 nm wavelength (Figure 18i.2), the EPSC was below the threshold till the pulse width increased to 40 ms. With the 360 nm light stimulus, the EPSC increased sharply upon increasing the spike duration (Figure 18i.3). The connection between spike duration and wound level is given by the wound factor (*η*), which computes the relation between the maximum EPSC signal and the pain threshold current. Figure 18i.4 demonstrates how the artificial nociceptor can recognize pain for different levels of injury by detecting changes in *η* based on spike duration.

Pain has both a physical and emotional component, and is linked to damage or the potential for damage to body tissue.^[^ ^38^ ^]^ Figure 18j.1 illustrates a child just pinched by a crab.^[^ ^38^ ^]^ Although the child is experiencing pain and fear, this situation can be considered a typical injury response or a low level of sensitization. However, if the exposure to a persistent and intense harmful stimulus takes place, the child would still experience discomfort even after moving the crab away. Such an effect is known as central sensitization and is illustrated in Figure 18j.2. The change from ordinary wound to severe pain with great sensitization can be mimicked by the photo‐sensitive EG‐VFET through Pavlovian instruction,^[^ ^189^ ^]^ as shown in Figure 18j.3.^[^ ^38^ ^]^ For the assays it is worth defining the sensitization degree factor (λ), provided by the successive relation between the EPSCs prompted during each spike pair after the instruction process. In the experiment of Su et al., ordinary wound correlates 0.0 ≤ λ < 0.4, mild central injury correlates 0.4 ≤ λ < 0.6, and severe 0.6 ≤ λ < 1.0.^[^ ^38^ ^]^ *V_G_* pulse pairs were employed to simulate the wound stimuli, while optical spike pairs stimulated the ITO channel to memorize the pain input. In Figure 18j.3, the EPSC activated by the subsequent electrical pulse in the spike pair results λ < 0.4, showing an ordinary wound was caused. Subsequently, the optical spikes with 0.4 ≤ λ < 0.6 suggested an increase in pain perception. Both light and electrical pulses were applied to the EG‐VFET following a fivefold instruction protocol to assess the correlation between pain input and memory (Figure 18j.3). With 0.71 > λ > 0.74, the findings indicated that the associative features of pain stimuli and memory may influence central sensitization.^[^ ^9^, ^36^ ^]^ The effect of instruction was assessed by the application of a second identical electrical spike pair. The EPSCs prompted by this second input were superior to the threshold current Figure 18j.3), indicating that adverse feelings may have considerable importance in the aching perception augmentation.^[^ ^190^ ^]^ This result is in line with reports from chronic patients who attest to the still‐existing painful sensations that end up forming pain memories.^[^ ^191^ ^]^ After instruction, the fifth electrical pulse pair remained superior to the threshold, whereas λ  =  0.47 implied the profound correlation between pain input and memorization.^[^ ^38^ ^]^ Noteworthy, such findings provide clear evidence of the potential to integrate artificial pain perception, memory, and sensitization in electronic applications. As well, the device can enhance pain‐sensitization through Pavlovian training using light stimulus, offering a valuable and unprecedented opportunity for multi‐dimensional pain assessment with cortical reorganization emulation.^[^ ^38^ ^]^ This advancement is significant for the development of new bio‐inspired electronics, including bionic apparatuses and intelligent biomedical tools.

**Figure 19:**
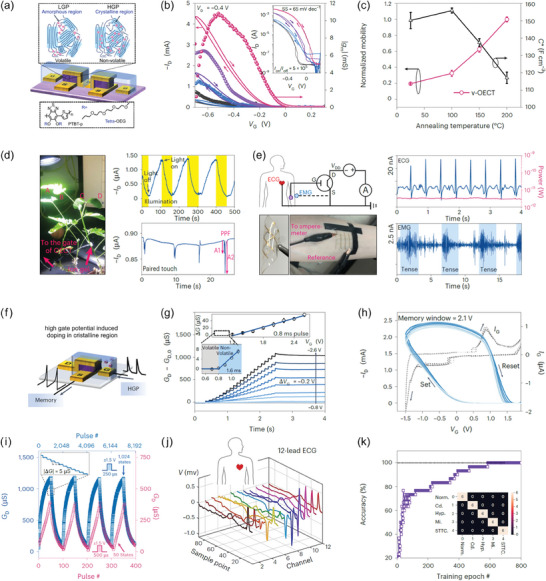
VOECT artificial synapse for multi‐modal sensing, memory, and processing. a) VOECT architecture sketch. The upper dashed box illustrates the ion contribution in the volatile and non‐volatile modes. The bottom dashed box exhibits the chemical structure of PTBT‐*p*. b) Transfer curves and transconductance acquired at different channel annealing temperatures. The pink, purple, and blue curves were obtained for channels annealed at 200, 150, and 100°C, respectively, whereas the black curve was for the as‐cast layer. The inset shows *SS* and *I*_ON/OFF_. c) Normalized *µ* and *C* ^*^ for the transistors. d) *I_D_* on time after submitting the *Mimosa pudica* plant to light and paired touch. The PPF behavior was recorded. e) Circuit diagram and experimental setup to acquire ECG/electromyography (EMG) signals using VOECTs. f) Illustration of the VOECT for a non‐volatile synapse operation. g) Influence of the *V_G_* pulse amplitude on non‐volatile conduction of the VOECT. h) Cyclic transfer curves for the non‐volatile mode of the VOECT acquired using high *V_G_*. i) LTPot curves for VOECT under controlled potential pulses. j) Real‐time heart disease diagnoses performed by a VOECT array. k) The simulated recognition accuracy of five types of ECG signals was analyzed for 800 training epochs. The classification confusion matrix post‐training is displayed in the inset. Adapted with permission.^[^ ^10^ ^]^ Copyright 2023, Wang et al. Published by Springer Nature.

Furthermore, VOECTs operating as a volatile receptor or a non‐volatile synapse providing sensing, memory, and processing functions can be achieved by managing the ion doping of the device, controlling its architecture, channel crystallinity, and electrode process.^[^ ^10^ ^]^ Wang et al. developed VOECTs featuring a crystalline‐amorphous PTBT‐*p* channel gated by an [EMIM^+^][TFSI^−^]:PVdF‐HFP ion gel or aqueous solution (**Figure** **19**a).^[^ ^10^ ^]^ The *d* value was 100 µm, and the vertical architecture allowed achieving *L* in the 40–80 nm range. The high *d*/*L* ratio (≈2000) was important for improving the amplification capability in the volatile mode setting, as it can minimize electrical potential gradients across the channel thickness and can maintain ions trapped in the PTBT‐*p* layer after the removal of the *V_G_*.^[^ ^10^ ^]^

The volatile property of the VOECTs as a multi‐modal sensing receptor (vision, gustation, and temperature sensation) was investigated by applying a low *V_G_*. Figure 19b shows the volatile transfer curves acquired for PTBT‐*p* channels with different degrees of crystallinity. Figure 19b,c shows that the transconductance and *µC* ^*^ increased for devices with higher degrees of crystallinity, indicating an improvement in the VOECT signal amplification capability that is important for sensing performance. The 200 °C‐annealed VOECT exhibited *g*_m_/*V_D_*  =  27 mS V^−1^, *I*_ON/OFF_  =  5 × 10^5^, *SS*  =  65 mV dec^−1^, and a stable volatile switching time of 6.67 ms for a 10^4^ µm^2^ device. These features indicated that the VOECTs were suitable for multi‐modal sensing. Then, Wang et al. monitored ion concentration changes in plants led by light and mechanical stimuli (Figure 19d), performed an ECG recording with a low energy consumption < 1 µW (Figure 19e),^[^ ^130^ ^]^ and demonstrated multi‐modal sensory ANNs for gustation, temperature, and light.^[^ ^10^ ^]^

The non‐volatile synaptic VOECT operated with a high applied *V_G_* with pulse amplitudes >|−0.8| V (Figure 19f), which modulated conductance changes in the channel, as shown in Figure 19g. The transfer curve with a centrosymmetric hysteresis in Figure 19h ensured the non‐volatile retention with a 2.1 V window.^[^ ^192^ ^]^ The LTPot characteristics in Figure 19i show 1024 states for the VOECT measured over an extensive range of dynamic conductance. The number of states can be set by varying the pulse width or amplitude. The *NL* was 0.20/1.63, as estimated from the LTPot/Dep curve acquired using 50‐state programming, whereas the signal‐to‐noise level was 179. Besides, 2000 current LTPot/Dep pulses in 50 cycles were also applied leading to a signal‐to‐noise level of ≈290 and a low cycle‐to‐cycle variation of ≈0.49%. The proposed VOECTs exhibited promising features for analog in‐memory computing, such as long retention, low switching stochasticity, a controlled‐vast range of states, and high‐speed pulse operation. Due to these attributes, they are promising candidates for processing sensory information collected from receptors in real time.^[^ ^10^ ^]^

In their experiments, Wang et al. also utilized STDP using two VOECTs arranged in a 1‐transistor‐1‐resistor (1T1R) configuration.^[^ ^10^ ^]^ The implementation of STDP on hardware led to non‐volatile and analog conductance fine‐tuning that is compatible with promising artificial synapses.^[^ ^193^ ^]^ In addition, the transistor channel had a high OFF‐state resistance that reduced the risk of *G* current leading to conductance drift.^[^ ^194^ ^]^ Furthermore, a homogeneous and biologically plausible spiking neural network^[^ ^195^ ^]^ can be obtained by employing the VOECTs developed by Wang et al. without heterogeneous integration or complex pulse engineering.^[^ ^196^ ^]^ In addition, the 1T1R system could also provide the network with the characteristics of an ANN. The simulated performance of a single‐layer spiking neural network or ANN for classifying MNIST handwritten digits was compared with the spiking neural network or ANN using resistive random‐access memories. Accordingly, the experimental *NL*, the cycling stability, and the device‐to‐device reproducibility of the VOECT arrangements were considered for the comparison. The classification accuracy for the spiking neural network was ≈89%, and for ANN was ≈91%, whereas for the resistive random‐access memory‐based spiking neural network was ≈83% and for the resistive random‐access memory‐based ANN ≈87%.^[^ ^10^ ^]^

Finally, Wang et al. performed real‐time diagnoses for heart diseases using reservoir computing composed of identical VOECTs.^[^ ^10^ ^]^ Figure 19j shows the disease ECG signals collected by the VOECT array. The system also serves as the neurons and computing nodes of a dynamic reservoir. Thus, the responses of experimentally calibrated devices were used to train a VOECT‐based ANN. Figure 19k shows that the accuracy of the simulated diagnoses was 100% after 700 training epochs, demonstrating the efficiency of the ANN for health applications. In addition, the detections can be extended to body fluid and temperature monitoring, and virus detection.

## 5 Critical Roadmap to Advance IGVT Neuromorphic Research

Despite the significant development of IGVTs achieved recently, further exploration is necessary for the research on IGVT‐based artificial synaptic systems. The interdisciplinary nature of this matter demands coverage of various areas, including solid‐state physics, material science, electronics, electrochemistry, and polymer chemistry. Even with a great deal of research already running, we believe that much effort is still required to exploit the real potential of synaptic IGVTs‐ viz., to boost power efficiency, miniaturization, low cost and integration with mass production technologies, and compatibility with complementary circuits and multi‐modal systems.^[^ ^43^ ^]^ In these scenarios, ionic liquid electrolytes can be a limitation in terms of the practical applicability of IGVTs. Liquid electrolytes are subject to leakage, evaporation, or electrolysis, compromising the long‐term performance and stability of devices.^[^ ^197^ ^]^ During the characterization of complementary circuits or the liquid‐electrolyte integration in multi‐IGVT arrays such as active pixels on a chip, for instance, liquid can influence or even disrupt the response of other nearby devices by connecting them incorrectly. If the same ionic liquid is in contact with multiple active IGTs, it does not matter whether each device features individually patterned semiconductor channels connected to their own *S* and *D* and/or switched by dedicated *G* electrodes, as the current modulation of one IGT will be affected by the neighboring device switching. Therefore, it is essential to be careful and precise in positioning the electrolyte only on the active part, as exemplified by Huang et al.^[^ ^54^ ^]^ Furthermore, the surface tension frequently makes the integration of multiple liquid components with miniaturized devices a complicated process, in addition to the fact that incorporating wet chemistry into final products (e.g., for commercialization) is not straightforward. Thereby, the development of patternable electrolytes offers an interesting prospect to enable the exploitation of IGVTs in high‐density integrated circuits and active‐matrix networks in dynamically morphing (viz. 4D) electronics.^[^ ^68^ ^]^ Recent studies have already addressed these issues in part by employing patternable solid electrolytes, which are also suitable for advancing wearable electronic sensing technologies.^[^ ^198^ ^]^

It is worth pointing out that more fundamental assessments are required to draw the picture of the ion migration to and through the electrolyte/semiconductor interface, and its influence on the IGVT's mixed ionic‐electronic transport. Researchers should employ different experimental and theoretical tools and consider many process‐dependent parameters to gain a better understanding of the ion‐gating principles. Following this direction, for example, Keene et al. have recently reported insightful findings about hole‐limited electrochemical doping in mixed ionic‐electronic conductors.^[^ ^199^ ^]^ They showed the assumption that electrochemical doping should be limited by ionic motion, due to ions weighing more than electrons and holes, does not hold for conjugated polymers at all.^[^ ^199^ ^]^ Further than enabling a more accurate design of conjugated polymers for electrochemical doping applications, their findings also shed light on the innovative experimental approaches that can be employed to improve the comprehension of the ion gating phenomena.^[^ ^200^ ^]^ Therefore, we confirm and stress that the IGVT technology can already provide a suitable hall to evaluate the mixed conduction properties of materials effectively at the nanoscale experimentally.

Similar to the astute contribution of Shahi et al.,^[^ ^201^ ^]^ where the authors have sympathetically discussed the long‐standing conundrum surrounding the µC* values assessed using electrochemical transistors, we argue here that the standardization of the IGVT figures‐of‐merit is another critical aspect to be improved in the near future. This claim arises from the fact that, compared with conventional (planar) IGTs, many vertical device architectures provide distinct boundary conditions for the material electric‐field distributions, the electrolyte ion transport, and the channel mixed‐ionic‐electronic transport. Consequently, standardizing the IGVT figures‐of‐merit implies that researchers should also invest time to refine the Bernards and Malliaras model^[^ ^63^ ^]^ to better outline the physics of ion gating in the extraordinary IGVT architectures. Such refined approaches will certainly provide more comprehensive insights into the latest achievements regarding power efficiency, memory retention, and plasticity of IGVTs, paving a consistent way for future achievements in this field.

Moving forward to the artificial synaptic applications, especially when they come to multi‐modal synaptic networks, our vision is that a robust theoretical bridge between the biological‐ and the IGT‐based synapses, built by vigorous computational simulations, is still lacking. First‐principle computational calculations are necessary to advance the current knowledge and provide stand‐alone models for the biological‐ and artificial processes involved with neuromorphic learning and plasticity. Notably, the IGVT technology offers tremendous potential to face this challenge by enabling the realization of sub‐10 nm devices that can be brute‐force computed by standard clusters these days, facilitating the consensus between theory and experiment. Thus, density‐functional‐theory and molecular‐dynamics simulations should be utilized to uncover the groundbreaking chemical‐physical properties of IGVT‐driven experimental assessments, whereas machine‐learning tools can be applied along with these approaches to predict novel materials and heterostructures to improve the IGVT neuromorphic functions‐ as successfully realized in other material research fields.^[^ ^202^, ^203^, ^204^ ^]^ We anticipate that by extending their scope to encompass the mixed ionic‐electronic charge transport in IGVTs, these robust computational methodologies will aid in identifying the superior materials for future high‐end applications of artificial synapses. With the development of this field, the IGVT technology will indeed assist researchers and technologists in centering their attention on deeply understanding and mapping the dynamic neural spike information computing functions to multi‐modal neuromorphic applications.

The human brain can process various stimuli simultaneously, including visual, auditory, tactile, gustatory, and olfactory inputs, body balance and awareness, and physiological pain stimulations. However, till now most IGVT artificial synapses have only focused on the activation by a single input or a few stimuli. We expect that, in the future, researchers will dedicate more efforts in this area to develop multi‐modal artificial synapses to better replicate the simultaneous brain functions accurately. The pioneering demonstrations on multisensory and multi‐modal synaptic IGVTs by G. Liu et al.,^[^ ^56^ ^]^ Y. Liu et al.,^[^ ^181^ ^]^ Wang et al.,^[^ ^10^ ^]^ and Su et al.^[^ ^38^ ^]^ do offer a splendid vista toward future developments of artificial synapses. The use of the IGVT technology in these implementations is a promising option due to the evident size reduction, the sub‐micrometer footprint, and the lower energy consumption compared to planar IGTs‐ besides the highlighted possibilities to perform electrical and ionic switching, mixed‐ionic‐electronic conduction, thermoelectric conversion, and photogating. Still, we are aware that the more independent working principles implemented in the same active unit, the greater the chance that multi‐modal functionality integration will be successful. Therefore, we enthusiastically expect that the integration of synaptic IGVT technology with spintronics, owing to the IGVT's architecture compatibility with the vertically assembled spin‐valve heterostructures,^[^ ^205^, ^206^, ^207^ ^]^ will provide additional degrees of freedom for the understanding and optimization of STP and LTP in multi‐modal applications. This ultimate vision holds the rationale and the means to inaugurate a new field of research between spintronics and flexible electronics, one that can be boosted by a novel IGVT‐based neuromorphic spintronic device technology.

## 6 Summary and Outlook

In this review, we provided a comprehensive discussion of the pioneering developments in IGVTs and their promising applications in integrated circuits and neuromorphic systems. We highlighted the similarities between IGVTs and biological synapses and also gave an overview of the device's technological evolution as artificial synaptic systems with various types of perception applications. The achievements in IGVTs show these devices can effectively emulate the behavior of biological synapses, displaying spike‐timing‐dependent plasticity − viz. a key property that allows neuromorphic systems to learn and adapt from input patterns. Such benefits make IGVTs viable for a variety of applications, including flexible electronics, bioelectronics, robotics, and brain‐inspired computing. In these scenarios, IGVTs can be considered game changers mostly due to their low power consumption, which is similar to or even lower than that of biological systems. Furthermore, IGVTs are harmonious with several biocompatible materials and can emulate the ion‐transport phenomena of biological synapses using a sub‐micrometer footprint. We foresee that IGVT research will stimulate the exploitation of ion gating at the nanoscale, providing advancements that shall span from new theoretical implementations to novel experimental realizations. Thereby, our critical roadmap shows that interdisciplinary research efforts should be put together to face the most standing challenges, viz. 1) the improvement of patternable‐electrolyte technologies, 2) the experimental and theoretical elucidation of the mixed ionic‐electronic conduction in vertical transistors, 3) the implementation of first‐principle methods to compute the mixed charge transport in nanoscale IGVTs, 4) the use of machine‐learning approaches to predict new functional materials for IGVT applications, and 5) the development of multi‐modal technologies. Our arguments draw attention to the IGVT's multi‐parametric properties as a remarkable tool to control at the sub‐micrometer scale, the electrical and ionic switching, the mixed‐ionic‐electronic conduction, the thermoelectric energy conversion, the photocurrent generation, and the spin‐dependent transport of several functional materials. We believe that IGVT‐based artificial synapses hold the potential to shake up the next‐generation multi‐modal technologies in flexible electronics, bioelectronics, spintronics, robotics and micro‐robotics, and neuroprosthetics. We expect that the progress discussed in this review will inspire the development of affordable neuromorphic technologies for comfortable living in our future smart digital world.

## Author Contributions

All the authors contributed meaningfully to the writing and revision. Review article conceptualization: LM.

## Conflict of Interest

The authors declare no conflict of interest.
